# Deciphering Genomic Underpinnings of Quantitative MRI-based Radiomic Phenotypes of Invasive Breast Carcinoma

**DOI:** 10.1038/srep17787

**Published:** 2015-12-07

**Authors:** Yitan Zhu, Hui Li, Wentian Guo, Karen Drukker, Li Lan, Maryellen L. Giger, Yuan Ji

**Affiliations:** 1 Program of Computational Genomics & Medicine, NorthShore University HealthSystem, Evanston, Illinois, USA; 2 Department of Radiology, The University of Chicago, Chicago, Illinois, USA; 3 School of Public Health, Fudan University, Shanghai, P.R. China; 4 Department of Public Health Sciences, The University of Chicago, Chicago, Illinois, USA

## Abstract

Magnetic Resonance Imaging (MRI) has been routinely used for the diagnosis and treatment of breast cancer. However, the relationship between the MRI tumor phenotypes and the underlying genetic mechanisms remains under-explored. We integrated multi-omics molecular data from The Cancer Genome Atlas (TCGA) with MRI data from The Cancer Imaging Archive (TCIA) for 91 breast invasive carcinomas. Quantitative MRI phenotypes of tumors (such as tumor size, shape, margin, and blood flow kinetics) were associated with their corresponding molecular profiles (including DNA mutation, miRNA expression, protein expression, pathway gene expression and copy number variation). We found that transcriptional activities of various genetic pathways were positively associated with tumor size, blurred tumor margin, and irregular tumor shape and that miRNA expressions were associated with the tumor size and enhancement texture, but not with other types of radiomic phenotypes. We provide all the association findings as a resource for the research community (available at http://compgenome.org/Radiogenomics/). These findings pave potential paths for the discovery of genetic mechanisms regulating specific tumor phenotypes and for improving MRI techniques as potential non-invasive approaches to probe the cancer molecular status.

Precise cancer diagnosis and treatment rely on the integration of information from various sources, such as the phenotypic and genotypic profilings of tumors. Radiogenomics aims to integrate computer-extracted phenotypes from radiological imaging data with genomic data, providing an opportunity to investigate the association between the radiomic tumor phenotypes and the genomic measurements of the same tumors. Such a study may identify the genetic mechanisms that regulate the development of specific tumor phenotypes. Radiomic phenotypes that are highly correlated with important genomic biomarkers can potentially serves as diagnosis and prognosis tools for patient monitoring, and therefore augment the utility of radiological imaging as a non-invasive technology for cancer care.

Radiogenomics is a new scientific field with scarce applications. This is mainly due to the lack of data consisting of both imaging and genomic measurements on the same set of tumors. Nevertheless, a few recent studies have pioneered early endeavor. Studies in lung cancer^1^,^2^, head & neck cancer^2^, glioblastoma multiforme^3^, and clear cell renal cell carcinoma^4^ attempted to correlate tumor radiomic phenotypes with DNA mutations^4^, mRNA expressions^1^,^2^,^3^ and copy number variations^3^. For breast cancer, Yamamoto *et al.*^5^ collected Magnetic Resonance Imaging (MRI) data and gene expression data from 10 human tumors and correlated 26 imaging phenotypes defined by radiologists with the expressions of individual genes and gene sets. Mazurowski *et al.*^6^ extracted radiomic phenotypes based on 48 patients and discovered the phenotypes associated with the luminal B subtype of breast cancer. Based on 56 Estrogen Receptor Positive (ER+) breast cancers, Ashraf *et al.*^7^ used computationally derived radiomic phenotypes to predict the recurrence likelihood score defined by Oncotype DX, a validated gene expression assay including 21 selected genes. Agner *et al.*^8^ extracted quantitative radiomic features of 76 breast lesions and used them to differentiate the triple-negative breast cancer from other subtypes. In addition, *BRCA1/2* and *UGT2B* variations have been associated with computer-extracted radiomic phenotypes^9^,^10^,^11^.

Here, we report a comprehensive radiogenomic study of breast invasive carcinoma based on the integration of The Cancer Imaging Archive (TCIA)^12^ and The Cancer Genome Atlas (TCGA)^13^, two leading cancer research projects supported by the U. S. National Institutes of Health. We integrated the Dynamic Contrast Enhanced-MRI (DCE-MRI) data with the multi-platform genomic data for 91 primary breast tumors. Through an extensive investigation, we identified statistically significant associations between various genomic features and radiomic phenotypes in breast invasive carcinoma that have yet to be reported. Among the many novel findings, we discovered highly specific associations of radiogenomic features, which are potentially useful for (1) the imaging based diagnosis that can inform the genetic progress of tumor and (2) the discovery of genetic mechanisms that regulate the development of tumor phenotype. We believe that our study is the first of its kind that investigates the relationships between the multi-layer tumor molecular system and the various quantitative radiomic phenotypes of breast cancer.

## Results

### Summary of Associations between Genomic Features and Radiomic Phenotypes

**Figure 1:**
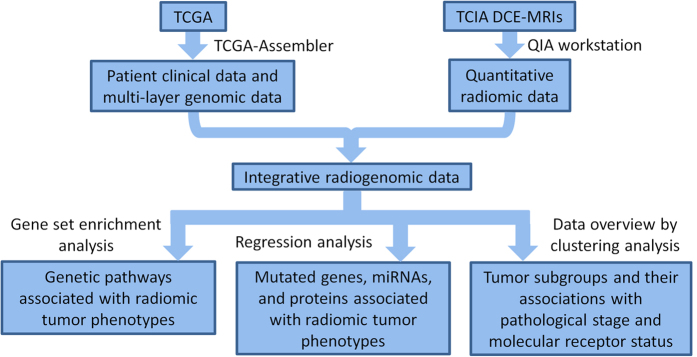
Flowchart illustrating the organization of data and analyses in the study. QIA refers to Quantitative Image Analysis.

**Figure 2:**
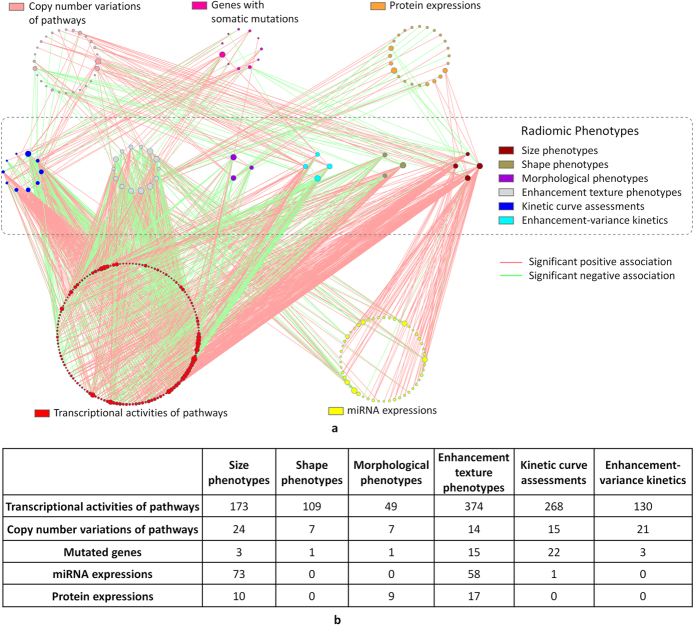
Overview of all identified statistically significant associations. (**a**) In the figure, each node is a genomic feature or a radiomic phenotype. Each line is an identified statistically significant association. Genomic features without statistically significant association are not shown. Genomic features are organized into circles by data platform and indicated by different node colors. Radiomic phenotypes are divided into six categories also indicated by different node colors. The node size is proportional to its connectivity relatively to other nodes in the category. Associations are deemed as statistically significant if the adjusted p-values ≤0.05. The only exception is for the associations involving somatically mutated genes, for which the statistical significance criteria are (1) p-value ≤0.05 and (2) the gene mutated in at least five patients. (**b**) A table showing the numbers of statistically significant associations between genomic features of different platforms and radiomic phenotypes of different categories.

Through the Gene-Set Enrichment Analysis (GSEA)^14^,^15^ (Supplementary Information Section 5) and the linear regression analysis (Supplementary Information Sections 6–8), we performed a quantitative study to associate genomic features, including miRNA expressions, protein expressions, and gene somatic mutations, and transcriptional activities and gene CNVs of all genetic pathways in the Kyoto Encyclopedia of Genes and Genomes (KEGG)^16^ database, with six categories of radiomic phenotypes, including tumor size, shape, morphology, enhancement textures, kinetic curve assessments, and enhancement-variance kinetics. The study schema is presented in Fig. 1 and the main results are presented in Fig. 2. Specifically, Fig. 2a shows the statistically significant associations and Fig. 2b summarizes the numbers of associations between different categories of genomic features and radiomic phenotypes. Fisher’s exact test^17^,^18^ was applied to the numbers reported in Fig. 2b and concluded that the frequencies of statistically significant associations are dependent on the categories of genomic features and radiomic phenotypes (p-value ≤1.0 × 10^−8^). In other words, some types of genomic features and radiomic phenotypes are more likely to be associated than others.

The most intriguing findings in Fig. 2 are related to the associations of two types of genomic features, 1) transcriptional activities of pathways and 2) miRNA expressions. Specifically, pathway transcriptional activities are associated with all six types of radimoic phenotypes with statistical significance (Table S3), indicating that they can regulate various aspects of the tumor phenotype. Strikingly, statistically significant associations between pathway transcriptional activities and all four tumor size phenotypes (including *lesion volume*, *effective diameter*, *surface area*, and *maximum linear size*) are extremely specific in that more than 97.7% of the associations are positive (adjusted p-values ≤6.21 × 10^−9^ by the Chi-squared proportion tests with equal proportions of positive and negative associations), indicating that many pathways are up-regulated during tumor growth since larger tumors are associated with mostly higher pathway activities. We find that pathway transcriptional activities are mostly negatively associated with two tumor morphological features including *margin sharpness* and *variance of radial gradient histogram* (with adjusted p-values ≤0.043 from the proportion tests). This suggests a positive correlation between the transcriptional activities of genetic pathways and a blurred tumor margin, which is potentially a sign of tumor invasion into the surrounding tissue. Also, the transcriptional activities of pathways are mostly positively associated with the irregularity of tumor shape, another sign of aggressive tumor, characterized by two radiomic phenotypes *irregularity* and s*urface to volume ratio* (with adjusted p-values ≤0.00285 from the proportion tests).

The associations between miRNA expressions and radiomic phenotypes are highly specific in that miRNA expressions are only associated with primarily two types of radiomic phenotypes, tumor size and enhancement texture (Fig. 2 and Table S3). Statistically significant associations between miRNA expressions and three out of the four tumor size phenotypes are dominantly positive with proportions ≥92.3% and adjusted p-values ≤0.00118 from the proportion tests. This suggests that miRNAs may mainly mediate the growth of tumor and the heterogeneity of blood vessel system in tumor. Such insights on the role of miRNA may facilitate the cancer mechanism study and the design of miRNA targeted treatment. Conversely, due to the specificity in the associated phenotypes, it is possible to use radiomic phenotypes characterizing the tumor size and enhancement texture to predict miRNA activities without the need for tumor biopsy and miRNA profiling.

Compared to the transcriptional activities of genetic pathways, the CNVs of pathways have much fewer statistically significant associations (Fig. 2), which are enriched with only the tumor size phenotypes (adjusted p-value = 7.95 × 10^−8^, Table S3) and the enhancement-variance kinetics (adjusted p-value = 4.46 × 10^−6^, Table S3). TCGA uses the Reverse Phase Protein Array (RPPA) to measure the expression levels of 142 proteins and phospho-proteins related to breast cancer. Protein expressions show enriched associations with the tumor size phenotypes (adjusted p-value = 0.0486, Table S3) and the morphological phenotypes (adjusted p-value = 0.0244, Table S3), but not with any other phenotype category.

We discuss the identified associations of different types of genomic features in detail in the next four sub-sections.

### Associations between Genetic Pathways and Radiomic Phenotypes

**Figure 3:**
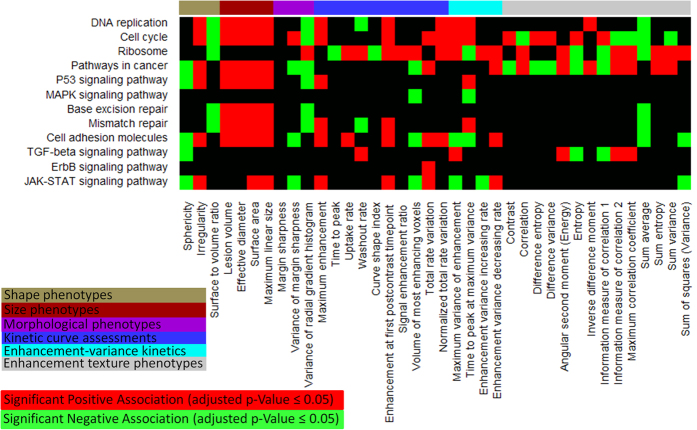
Heatmap representation of statistically significant associations between radiomic phenotypes and transcriptional activities of some cancer-related genetic pathways. In the heatmap, genetic pathways are rows and radiomic phenotypes are columns.

The associations between the transcriptional activities of KEGG pathways and the radiomic phenotypes were studied using GSEA^14^,^15^. A total of 1,103 statistically significant (adjusted p-values ≤0.05) associations have been identified (Fig. 2 and Table S4). Fig. 3 provides some examples involving cancer-related pathways, which we elaborate below.

#### Cell Cycle, DNA Replication and Ribosome

Tumor growth requires excessive cell proliferation, for which 1) DNA replication, 2) protein synthesis, and 3) cell cycle are essential. Genes involved in cell cycle and DNA replication are positively associated with all four tumor size phenotypes (Fig. 3), indicating their activations during tumor growth. All these three gene modules are also positively associated with *enhancement at the first post-contrast time point, normalized total rate variation,* and *maximum variance of enhancement*, which characterize the blood flow dynamics and the contrast uptake heterogeneity in tumor.

#### Conserved Regulations in Cancer

KEGG does not provide a genetic pathway dedicated to breast cancer, but provides a comprehensive regulation map called “pathways in cancer“^19^ that includes the conserved regulation mechanisms across cancer types. The transcription activity of this large molecular regulation system is positively associated with 14 radiomic phenotypes and negatively associated with 9 radiomic phenotypes (Fig. 3). It is positively associated with the tumor size phenotypes. Its activity is also associated with an increased tumor shape irregularity characterized by the *irregularity* and *sphericity* phenotypes, which is usually a sign of malignant and aggressive tumor^20^.

#### JAK-STAT Signaling Pathway

The JAK-STAT signaling cascade forms the principal signaling transduction mechanism in response to a variety of cytokines and growth factors^21^. Over-activation of the JAK-STAT pathway can cause cancer by evading apoptosis and forming self-sufficient growth signals^22^. According to our analysis, the transcriptional activity of JAK-STAT signaling pathway is positively associated with the tumor size phenotypes (Fig. 3). Also, we found a statistically significant association between its activity and the tumor shape irregularity measured by two radiomic phenotypes, *irregularity* and *sphericity*. We also see that the pathway has statistically significant positive associations with *maximum* *enhancement* and *enhancement at first post-contrast time point*, which implies that tumors with a higher JAK-STAT pathway activity have more leaky microvessels to support its growth.

#### Cell Adhesion Molecules

The cell adhesion molecules are in direct or indirect control of cellular activities such as adhesion, proliferation, migration and differentiation. Aberrant activities of cell adhesion molecules disrupt normal cell-cell and cell-matrix interactions and can facilitate tumor formation and metastasis^23^. In Fig. 3, we find that expressions of cell adhesion genes are correlated with the signs of tumor malignancy and aggressiveness, such as a large tumor size (measured by all four tumor size phenotypes), an increased tumor shape irregularity (characterized by *irregularity* and *sphericity*), and increased blood flow dynamics (measured by *maximum enhancement*, *enhancement at the first post-contrast time point*, and *uptake rate*).

#### TGF-beta Signaling Pathway

The role of TGF-beta signaling pathway in breast cancer has been intensively studied^24^,^25^. *TGF-beta* serves as a tumor suppressor at the initial stage of tumorigenesis, but loses its growth inhibition function during cancer progression and diverts towards promoting motility, invasion and metastasis at late stage^25^. We find that a strong activity of TGF-beta signaling pathway is positively associated with irregular tumor shape characterized by *sphericity* (Fig. 3). Its statistically significant positive association with the *maximum variance of enhancement* implies a correlation between the activity of TGF-beta signaling pathway and the heterogeneous blood distribution in tumor.

Section 5 in the Supplementary Information provides more explanations on the associations involving other cancer-related pathways shown in Fig. 3. We also identified statistically significant associations between the CNVs of pathways and radiomic phenotypes (Fig. S2 in the Supplementary Information Section 5). For example, the copy number amplification of JAK-STAT signaling pathway is associated with an increased tumor shape irregularity.

### Associations between miRNA Expressions and Radiomic Phenotypes

**Figure 4:**
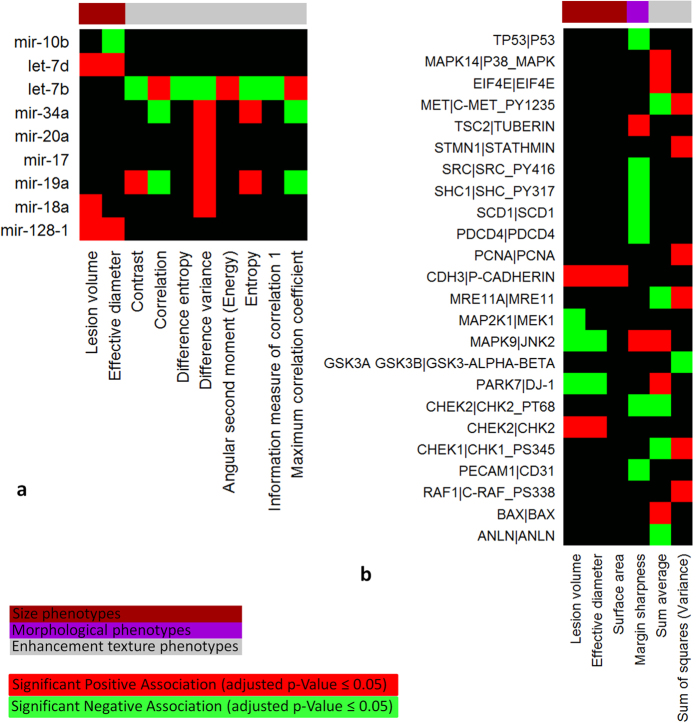
(a) Statistically significant associations between radiomic phenotypes and expressions of selected cancer-related miRNAs. Only radiomic phenotypes and miRNAs with statistically significant associations are shown. (**b**) **Statistically significant associations between radiomic phenotypes and RPPA protein expressions.** Only proteins and radiomic phenotypes with statistically significant associations are shown. For each protein, the name of the protein is shown after “|” and the gene that encodes the protein is shown before “|”.

Using the linear regression analysis (see Section 6 in the Supplementary Information), we found statistically significant (adjusted p-value ≤0.05) associations between miRNA expressions and primarily two types of radiomic phenotypes, tumor size and enhancement texture (Fig. 2). Table S5 in the Supplementary Information shows all the identified associations. We curated a list of miRNAs related to cancer development, especially breast cancer formation, by literature survey^26^,^27^,^28^,^29^, and present in Fig. 4a the statistically significant associations involving these cancer-related miRNAs. *MiR-128-1* plays an oncogenic role in drug-resistant breast cancer cell by interfering with TGF-beta signaling^27^. Its expression is found to be positively associated with the tumor size. *MiR-18a* has been reported to induce tumor growth and tumor vascularization^26^. Its expression is positively associated with *lesion volume* and *difference variance*. Both *miR-19a* and *miR-18a* belong to the miR-17-92 cluster. The expression of *miR-19a* is statistically significantly associated with enhancement texture phenotypes, including *contrast*, *correlation*, *difference variance*, *entropy*, and *maximum correlation coefficient*. These associations indicate that the expression of *miR-19a* correlates with the heterogeneity of tumor enhancement texture, which is also a sign of aggressive and malignant lesion^20^. *Let-7b* shows an opposite association pattern with the enhancement texture phenotypes compared to *miR-19a*, probably due to the tumor suppressive function of the let-7 family^26^. *MiR-10b* has been reported as a modulator of tumor invasion and metastasis^26^,^27^. Its expression is associated with tumor *effective diameter*.

### Associations between Protein Expressions and Radiomic Phenotypes

All statistically significant (adjusted p-value ≤0.05) associations between protein expressions and radiomic phenotypes are shown in Fig. 4b. P-cadherin is a calcium-dependent cell-cell adhesion glycoprotein encoded by *CDH3* in human. Its expression has been shown to be correlated with high histologic grade, increased proliferation, and poor patient survival in breast cancer^30^,^31^. We find a statistically significant positive association between the expression of P-cadherin and the tumor size, measured by *effective diameter*, *surface area*, and *lesion volume*. JNK2 is a mitogen-activated protein kinase encoded by *MAPK9* in the MAPK signaling pathway. It is considered as a negative regulator of cellular proliferation^32^, and cooperates with JNK1 in activating the p53 singling pathway to induce apoptosis^33^. The anti-tumorigenic role of JNK2 is demonstrated in our analysis as its expression is negatively associated with the tumor size phenotypes and positively associated with tumor *margin sharpness*, a phenotype signaling the absence of tumor invasion into the surrounding tissue.

### Associations between Somatic Gene Mutations and Radiomic Phenotypes

We compared the measurements of a radiomic phenotype for patients harboring somatic mutations in a gene versus those not (Section 8 in the Supplementary Information). Table S7 in the Supplementary Information shows all the associations in which a gene mutated in at least five patients and the obtained p-value ≤0.05.

*PIK3CA* is an oncogene participating in the signaling cascades of cell growth, survival, proliferation, motility and morphology. Four radiomic phenotypes, including one kinetic curve assessment and three enhancement texture phenotypes, are associated with *PIK3CA* mutations. *GATA3* has been observed by TCGA as the third most frequently mutated gene in breast invasive carcinoma after *TP53* and *PIK3CA*, with an overall mutation rate larger than 10%^13^. *GATA3* encodes a transcription factor that regulates luminal epithelial cell differentiation in the mammary gland^34^. Its expression is progressively lost during luminal breast cancer progression as cancer cells acquire a stem cell-like phenotype^35^. Our analysis shows that mutations in *GATA3* are negatively associated with tumor size (measured by three tumor size phenotypes), tumor shape irregularity (measured by *irregularity*), and *sum entropy* that measures the randomness of enhancement texture. Such an observation leads to the hypothesis that mutations in *GATA3*, although frequent, might not be driver mutations causing tumor progression, because a large tumor size, an irregular tumor shape, and random enhancement texture are usually signs of malignant and aggressive tumors. *MAP2K4* encodes a kinase in the MAPK signaling pathway and is considered as a tumor suppressor^36^. Its mutations are positively associated with *time to peak* and negatively associated with *uptake rate*, indicating tumors with *MAP2K4* mutations have a relatively slow blood flow and fewer leaky microvessels.

We also studied the associations of somatic gene mutations at the pathway level, by comparing the measurement of a radiomic phenotype for patients with gene mutations in a KEGG pathway versus those without (see Section 8 in the Supplementary Information). Table S8 in the Supplementary Information shows all the identified statistically significant associations. An interesting observation is that somatic mutations in the p53 signaling pathway was found to be positively associated with tumor *effective diameter*, indicating potentially that DNA mutations can damage the tumor suppressive function of the p53 signaling pathway and thus induce tumor growth.

## Discussion

Based on the integrated data from TCIA and TCGA, we have conducted a comprehensive radiogenomic study to explore the association between multi-platform genomic profiles and MRI-based tumor phenotypes for breast invasive carcinoma. Our study generated two major findings that have not been previously reported. First, we identified statistically significant associations between six types of radiomic tumor phenotypes and various genomic features involved in multiple molecular regulation layers. Our large-scale study produced a new resource (available at http://compgenome.org/Radiogenomics/) for exploring the genetic mechanisms that potentially regulate the formation of various tumor phenotypes. We expect our findings will facilitate future radiogenomic research and provide a template for the type of analyses that could be carried out. Second, we observed highly specific patterns for the identified associations. Many genetic pathways are more active in tumors with a large size, irregular shape, and blurred margin. MiRNA expressions are associated with only tumor size and enhancement texture, but not other types of radiomic phenotypes. These patterns provide new insights on the genetic mechanisms that regulate tumor development. They are also potentially useful in the clinical diagnosis of cancer by suggesting the candidate radiomic phenotypes for predicting genomic features, although further validations are needed. If validated, these findings could augment the use of MRI as a non-invasive technology, not only for examining tumor phenotypes, but also for probing the underlying molecular status of tumor, which is crucial for personalized treatment.

Compared to the associations at the transcriptional level, we found much fewer statistically significant associations for pathway CNVs and gene somatic mutations. There could be two reasons for this observation. Firstly, DNA mutation events, such as CNVs and somatic mutations are rarely shared across many patients (Table S6). Thus, there lacks sufficient statistical power for identifying a potential association, especially given the small sample size in almost all radiogenomic studies. We believe that our initial results can trigger the motivation for future large-scale radiogenomic studies that include a large number of samples. Secondly, compared to DNA mutations, gene expressions are more directly related to phenotype in the process of genetic events influencing phenotype development. Genetic mutations are more upstream in the functional activities of the cellular system. Therefore, gene expressions present more associations with tumor phenotypes.

An interesting finding is that the transcriptional activity of basal cell carcinoma pathway is statistically significantly associated with 12 radiomic phenotypes of breast invasive carcinoma (Table S9). Actually, among all the cancer-type-specific KEGG pathways, the basal cell carcinoma pathway is associated with the largest number of breast cancer radiomic phenotypes (Section 9 and Table S9 in the Supplementary Information). Such associations between breast and skin cancer are consistent with their correlation in terms of disease prevalence. Patients with basal cell carcinoma, especially those diagnosed at a young age, have been reported to have an increased risk for noncutaneous cancers including breast cancer^38^.

Our study is based on genomic data generated by a single tissue sample from each primary tumor. Tumor contains spatially heterogeneous cell populations. A single biopsy sample of a tumor usually contains multiple cell subpopulations, but typically cannot encompass all the subclones of a tumor. Therefore, the genomic profile of a single tumor sample may be incomplete and only partially reflect the overall genomic landscape of the entire tumor. All these can affect the results obtained through the radiogenomic analysis. A comprehensive study would require multiple biopsy samples from the same tumor, which is often costly and labor-intensive in practice. In return, such a study will be more informative and accurate.

In addition to the association analysis, we did clustering analysis on the tumor samples to provide an overview of the radiomic and expression data used in the analysis (Fig. S1 and Section 3 in the Supplementary Information). The clustering partitions of tumors were associated with the clinical subtypes of tumors defined by their pathological state and molecular receptor status (Table S2). Tumor clustering partitions based on gene expressions, miRNA expressions, and protein expressions are all statistically significantly (adjusted p-values ≤ 0.05) associated with the statuses of Estrogen Receptor (ER) and Progesterone Receptor (PR), which means that ER+ patients and PR+ patients show different expression patterns from ER − patients and PR − patients, respectively, at multiple molecular levels (Table S2 and Fig. S1).

Survival analysis is not included in our study, because of the short overall follow up (median is 870 days) and the small number of mortality events (1 out of 91). In this work, we are focusing on understanding the relationship between MRI tumor phenotypes and underlying genetic mechanisms, if there are any. We attempt to address a critical aspect of patient care – the use of noninvasive technology. Genomic features reflect the molecular characteristics of a tumor, but are obtained through invasive procedures such as surgery or biopsy. Through the study of radiogenomics, we aim to identify good surrogate radiomic features that can reveal genetic changes of tumors, thereby establishing noninvasive means for monitoring tumor progression.

More analyses have been planned for future radiogenomic study of breast cancer. Integration of multiple genomic, epigenomic, proteomic features simultaneously with radiomic phenotypes can provide a better understanding of how the multiple molecular regulation layers generate the observed tumor phenotypes. Graphical models can be a powerful tool to study the complex relationships between radiomic and genomic features^39^, which takes into account the potential competitive regulations and conditional dependence between them. Another interesting topic is to use predictive modeling to predict the status of a genomic feature (especially the biomakers important for diagnosis, prognosis, and response to therapy) based on imaging phenotypes that have been shown to associate with genomic features.

## Methods

The preparation of data and the organization of the analysis in this study are illustrated in Fig. 1. The DCE-MRIs of 91 breast cancers were downloaded from TCIA. These cases were contributed by four institutions, including Memorial Sloan Kettering Cancer Center, Mayo Clinic, University of Pittsburg Medical Center, and Roswell Park Cancer Institute. Section 1 in the Supplementary Information introduces the imaging cases, including the patient populations and the MRI pulse sequences used. Using a quantitative MRI radiomics workstation, i.e. the Quantitative Image Analysis (QIA) workstation^20^,^40^,^41^,^42^,^43^,^44^,^45^ that had been initially developed for computer-aided diagnosis research, we computationally extracted 38 radiomic phenotypes from the DCE-MRIs for each of the 91 primary tumors to characterize size, shape, margin, enhancement texture, kinetics, and variance kinetics. See Table S1 in the Supplementary Information for a summary of the radiomic phenotypes by category. Section 1 in the Supplementary Information also provides the mathematical descriptions of radiomic phenotypes. Using TCGA-Assembler^46^, we retrieved and processed the multi-layer genomic data of these tumors from TCGA, including gene expressions, copy number variations (CNV), protein expressions, miRNA expressions, and somatic mutations. Section 2 in the Supplementary Information introduces the sample inclusion criteria of TCGA and the assays, platforms, and algorithms used for generating the genomic data.

The radiomic data and genomic data were then combined as described in Fig. 1. See Table 1 for a summary of the combined radiogenomic data. To provide an overview of the radiogenomic dataset, unsupervised clustering on tumor samples was performed to identify tumor subgroups defined by individual data platforms (Fig. S1). The obtained tumor clusters were then associated with the tumor pathological stage and molecular receptor status (see Table S2). Details of the clustering analysis and results can be found in the Supplementary Information Section 3. Section 4 of the Supplementary Information tests the enrichment of the identified associations for each category of genomic features and radiomic phenotypes, with the results shown in Table S3.

We used the R package PIANO^15^ to perform the GSEA for identifying the associations between radiomic phenotypes and genetic pathways. Section 5 in the Supplementary Information introduces the PIANO package, its parameter setting, and other details of the analysis. Table S4 shows the median adjusted p-values for all associations between the radiomic phenotypes and the pathway transcriptional activities. Fig S2 is a heatmap presentation of all statistically significant associations between the radiomic phenotypes and the copy number variations of pathways. Additional discussions on the associations involving transcriptional activities of cancer-related pathways are included in Section 5 of the Supplementary Information.

Sections 6, 7 and 8 of the Supplementary Information introduce the details of linear regression analysis for identifying associations between radiomic phenotypes and three types of genomic features, including miRNA expression, protein expression, and gene somatic mutation, respectively. Data preprocessing steps, mathematical formulas, and the analysis procedure are included in those sections. Table S5 shows the analysis results of all identified associations involving miRNA expressions. Table S6 summarizes the frequencies of somatic gene mutations among patients. Table S7 and Table S8 present the analysis results of all identified associations involving somatic gene mutations at the single gene level and at the pathway level, respectively.

Section 9 and Table S9 in the Supplementary Information summarize the numbers of associations between the radiomic phenotypes of invasive breast carcinoma and the transcriptional activities of KEGG pathways dedicated to other types of cancer. This information is provided for the discussion on the relationship between breast cancer and basal cell carcinoma.

## Additional Information

**How to cite this article**: Zhu, Y. *et al.* Deciphering Genomic Underpinnings of Quantitative MRI-based Radiomic Phenotypes of Invasive Breast Carcinoma. *Sci. Rep.* **5**, 17787; doi: 10.1038/srep17787 (2015).

## Supplementary Material

Supplementary Information

Supplementary Table S4

## Figures and Tables

**Table 1:**
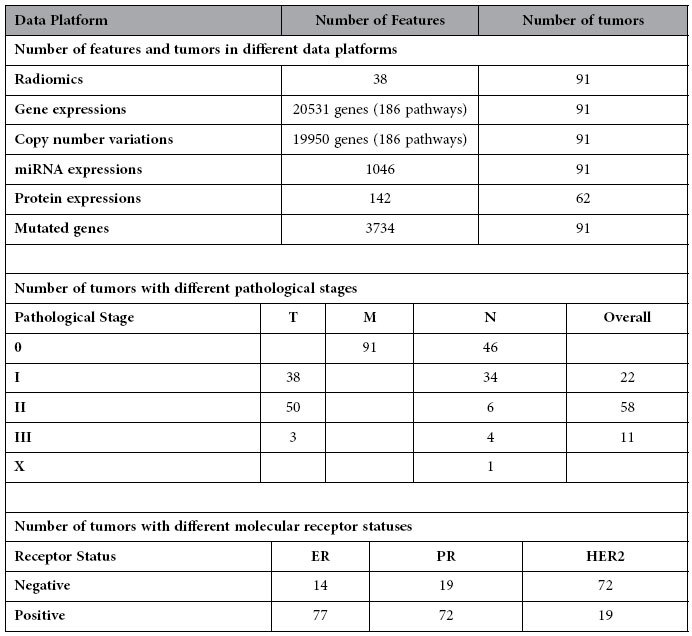
Summary of integrated data.

ER, PR and HER2 refer to Estrogen Receptor, Progesterone Receptor, and Human Epidermal growth factor Receptor 2, respectively.
