# Supplementary material for: Deciphering Genomic Underpinnings of Quantitative MRI-based Radiomic Phenotypes of Invasive Breast Carcinoma
Source: Sci Rep. 2015 Dec 7;5:17787. doi: 10.1038/srep17787 (PMC4671006; doi:10.1038/srep17787)
Supplement: Supplementary Information [file srep17787-s1.pdf]

Deciphering Genomic Underpinnings of Quantitative MRI-based Radiomic Phenotypes of Invasive Breast Carcinoma

(Supplementary Information)

Yitan Zhu[§1], Hui Li[§2], Wentian Guo[3], Karen Drukker[2], Li Lan[2], Maryellen L. Giger*[2] , Yuan Ji*[1,4]

1. Program of Computational Genomics & Medicine, NorthShore University HealthSystem, Evanston, Illinois, USA
2. Department of Radiology, The University of Chicago, Chicago, Illinois, USA
3. School of Public Health, Fudan University, Shanghai, P.R. China
4. Department of Public Health Sciences, The University of Chicago, Chicago, Illinois, USA

§ Equal contribution authors
* Co-corresponding authors

Contents

Section 1. Preparation of Radiomic Data 3

Section 2. Preparation of Genomic Data 11

Section 3. Overview of Radiogenomics Data 11

Section 4. Overview of Identified Statistically Significant Associations 14

Section 5. Associations between Genetic Pathways and Radiomic Phenotypes 15

Section 6. Associations between miRNA Expressions and Radiomic Phenotypes 18

Section 7. Associations between Protein Expressions and Radiomic Phenotypes 24

Section 8. Associations between Somatic Gene Mutations and Radiomic Phenotypes 25

Section 9. Associations between Non-BRCA Cancer Pathways and BRCA Radiomic Phenotypes 29

References 29

Section 1. Preparation of Radiomic Data

The MR images used in this study were downloaded from The Cancer Imaging Archive (TCIA, http://www.cancerimagingarchive.net). There were 108 MRI cases available at the time of this study. In order to minimize the variations from different imaging acquisition protocols, only 93 MRI cases that were acquired on a 1.5 Tesla magnetic strength with GE scanners were included. By excluding one case with missing images in the dynamic sequence and one case without genomic data, a total of 91 cases were used in the final dataset. All cases were primary tumors from female patients. The average age of patients was 53.6 years with a standard deviation of 11.5 years and ranging from 29 to 82 years with a median of 53 years. Out of the 91 invasive breast carcinoma cases, 79 were ductal carcinoma, 10 were lobular carcinoma, and 2 were mixed. Table 1 in the main text summarizes the 91 cases and their tumor pathological stages and molecular receptor status. These cases were contributed by four institutions: Memorial Sloan Kettering Cancer Center, Mayo Clinic, University of Pittsburg Medical Center, and Roswell Park Cancer Institute. MR images acquired from a dynamic-contrast enhanced T1-weighted protocol were used in this study. In general, there are one pre-contrast, and three to five post-contrast images obtained using a standard double breast coil on a 1.5T GE whole-body MRI system with T1-weighted 3D spoiled gradient echo sequence and a Gadolinium-based contrast agent. In-plane resolution ranged from 0.53 to 0.86 mm, and spacing between slices ranged from 2 to 3 mm.

Each MRI case was independently reviewed by 3 of 11 expert breast radiologists who were blinded to the clinical outcomes. The primary tumor location was established by their consensus and was the only input to the computer for the subsequent quantitative image analysis. Using this lesion location, each primary breast tumor was automatically segmented from background parenchyma using a fuzzy c-means clustering method[1]. This segmentation yields the tumor margin. Next, using our quantitative image analysis (QIA) MRI workstation (from computer-aided diagnosis), a total of 38 mathematical descriptors of the breast tumors were automatically extracted. These descriptors can be divided into six MRI phenotypic categories describing the tumor (1) size, (2) shape, (3) morphology, (4) enhancement texture, (5) enhancement kinetic curve, and (6) variance of enhancement kinetics[2-7]. Table S1 summarizes the formal name, brief description, and category of all radiomic phenotypes.

Table S1 List of names, descriptions, and categories of all 38 radiomic phenotypes.

Category	Name	Description
**Size phenotypes**	Lesion volume (mm^3)	Volume of lesion
	Effective diameter (mm)	Diameter of a sphere with the same volume as the lesion
	Surface area (mm^2)	Lesion surface area
	Maximum linear size (mm)	Maximum distance between any two voxels in the lesion
**Shape phenotypes**	Sphericity	Similarity of the lesion shape to a sphere
	Irregularity	Deviation of the lesion surface from the surface of a sphere
	Surface to volume ratio (1/mm)	Ratio of surface area to volume

**Morphological phenotypes**	Margin sharpness	Mean of the image gradient at the lesion margin
	Variance of margin sharpness	Variance of the image gradient at the lesion margin
	Variance of radial gradient histogram	Indicates how well the enhancement structure in a lesion extends in a radial pattern originating from the center of the lesion
**Enhancement texture phenotypes**	Angular second moment (Energy)	Measure of image homogeneity
	Contrast	Measure of local image variations
	Correlation	Measure of image linearity
	Entropy	Measure of the randomness of the gray-levels
	Sum of squares (Variance)	Measure of the spread in the gray-level distribution
	Difference entropy	Measure of the randomness of the difference of neighboring voxels' gray-levels
	Difference variance	Measure of variations of difference of gray-levels between voxel-pairs
	Inverse difference moment	Measure of the image homogeneity
	Sum average	Measure of the overall image brightness
	Sum entropy	Measure of the randomness of the sum of gray-levels of neighboring voxels
	Sum variance	Measure of the spread in the sum of the gray-levels of voxel-pairs distribution
	Information measure of correlation 1	Measure of nonlinear gray-level dependence
	Information measure of correlation 2	Measure of nonlinear gray-level dependence
	Maximum correlation coefficient	Measure of nonlinear gray-level dependence
**Kinetic curve assessments**	Maximum enhancement	Maximum contrast enhancement
	Time to peak (s)	Time at which the maximum enhancement occurs
	Uptake rate (1/s)	Uptake speed of the contrast enhancement
	Washout rate (1/s)	Washout speed of the contrast enhancement
	Curve shape index	Difference between late and early enhancement
	Enhancement at first postcontrast timepoint	Enhancement at first postcontrast timepoint
	Signal enhancement ratio	Ratio of initial enhancement to overall enhancement
	Volume of most enhancing voxels (mm^3)	Volume of the most enhancing voxels
	Total rate variation ($1/s^2$)	Measures how rapidly the contrast will enter and exit from the lesion
	Normalized total rate variation ($1/s^2$)	Measures how rapidly the contrast will enter and exit from the lesion
**Enhancement-variance kinetics**	Maximum variance of enhancement	Maximum spatial variance of contrast enhancement over time
	Time to peak at maximum variance (s)	Time at which the maximum variance occurs
	Enhancement variance increasing rate (1/s)	Rate of increase of the enhancement-variance during uptake
	Enhancement variance decreasing rate (1/s)	Rate of decrease of the enhancement-variance during washout

Group 1: Size phenotypes

1. Lesion volume (V):

Volume of the lesion, denoted by V

2. Effective diameter (d):

Diameter of a sphere with the same volume as the lesion

$$d = 2\sqrt[3]{\frac{3V}{4\pi}}$$

where V is lesion volume

3. Surface area (S):

Lesion surface area, denoted by S

4. Maximum linear size:

Maximum distance between any two voxels in the lesion

Group 2: Shape phenotypes[2]

1. Sphericity:

   Similarity of the lesion shape to a sphere

$$\text{Sphericity} = \frac{V_e}{V}$$

   where V_e denotes the volume of the lesion within a sphere of the effective diameter d, and V is lesion volume

2. Irregularity:

   Deviation of the lesion surface from the surface of a sphere

$$\text{Irregularity} = 1 - \frac{\pi d^2}{S}$$

   where d is the lesion effective diameter, and S is the lesion surface area

3. Surface area to volume ratio:

   Ratio of surface area to volume

$$\frac{\text{Surface area}}{\text{Volume}} = \frac{S}{V}$$

   where S is the lesion surface area, and V is the lesion volume

Group 3: Morphological phenotypes

Morphological tumor phenotypes describe combined shape and margin characteristics of the lesion[2].

1. Margin sharpness:

   Mean of the image gradient at the lesion margin

$$\text{Margin sharpness} = \frac{1}{N}\sum_{i=1}^{N} \|\nabla S(x_i, y_i, z_i)\|$$

where N is the number of voxels on the surface of the lesion, $S(x_i, y_i, z_i)$ is the signal intensity of the ith voxel located at (x_i, y_i, z_i) in the shell, and ∇ is the voxel-value gradient:

$$\|\nabla S(x_i, y_i, z_i)\| = \left\{ \begin{array}{c} \left(\frac{S(x_i+1, y_i, z_i) - S(x_i-1, y_i, z_i)}{2\delta_x}\right)^2 + \\ \left(\frac{S(x_i, y_i+1, z_i) - S(x_i, y_i-1, z_i)}{2\delta_y}\right)^2 + \\ \left(\frac{S(x_i, y_i, z_i+1) - S(x_i, y_i, z_i-1)}{2\delta_z}\right)^2 \end{array} \right\}^{1/2}$$

with δ_x, δ_y, δ_z denoting the voxel size in three dimensions in world coordinates.

2. Variance of margin sharpness:
Variance of the image gradient at the lesion margin

$$\text{Variance of margin sharpness} = \frac{1}{N-1}\sum_{i=1}^{N} (\|\nabla S(x_i, y_i, z_i)\| - \text{Margin sharpness})^2$$

3. Variance of radial gradient histogram:
Degree to which the enhancement structure extends to have a radial pattern originating from the center of the lesion

$$\text{Variance of radial gradient histogram} = \mathrm{var}\left(H(R_g)\right)$$

where $R_g = \frac{|\nabla[S_b(r)]\cdot(r-r_c)|}{\|\nabla[S_b(r)\|\cdot\|(r-r_c)\|}$ is the radial gradient value at location r and defined as the normalized dot product of the gradient direction and the radial direction vector, $S_b$denotes the voxel value at location r (r = (x,y,z)) in the 3D lesion, and r_c is the center of the lesion, $H(R_g)$ is the radial gradient histogram in a given of volume of interest.

Group 4: Enhancement texture phenotypes

Enhancement texture phenotypes characterize the textural properties of the contrast-enhanced tumors on the first post-contrast images, i.e., the heterogeneity of the uptake. These texture phenotypes were calculated from the gray-level co-occurrence matrix (GLCM)[3-4].

Some necessary notations are the following.
$p(i,j)$: the (i,j) entry of the normalized GLCM, which is a probability of two neighboring voxels, one with gray level i and the other with gray level j.
G: the number of distinct gray-levels in the image. GLCM is of the size G × G.
$p_x(i) = \sum_{j=1}^{G} p(i,j)$: the marginal row probabilities
$p_y(j) = \sum_{i=1}^{G} p(i,j)$: the marginal column probabilities

$$p_{x+y}(k) = \sum_{i=1}^{G}\sum_{j=1}^{G} p(i,j), \quad i + j = k, \quad k = 2,3,\ldots,2G$$

$$p_{x-y}(k) = \sum_{i=1}^{G}\sum_{j=1}^{G} p(i,j), \quad |i - j| = k, \quad k = 0,1,\ldots,G - 1$$

μ: the mean of $p(i,j)$.

μ_x: the mean of p_x

μ_y: the mean of p_y

μ_{x-y}: the mean of p_{x-y}

σ_x: the standard deviation of p_x

σ_y: the standard deviation of p_y

$HX = -\sum_{i=1}^{G} p_x(i)\log(p_x(i))$: the entropy of p_x

$HY = -\sum_{j=1}^{G} p_y(j)\log(p_y(j))$: the entropy of p_y

$HXY = -\sum_{i=1}^{G}\sum_{j=1}^{G} p(i,j)\log(p(i,j))$: the entropy of $p(i,j)$

$$HXY1 = -\sum_{i=1}^{G}\sum_{j=1}^{G} p(i,j)\log(p_x(i)p_y(j))$$

$$HXY2 = -\sum_{i=1}^{G}\sum_{j=1}^{G} p_x(i)\,p_y(j)\log(p_x(i)p_y(j))$$

1. Contrast:
   Contrast measures local image variations

$$\text{contrast} = \sum_{k=0}^{G-1} k^2 \left\{ \sum_{|i-j|=k} p(i,j) \right\}$$

2. Correlation:
   Correlation measures image linearity

$$\text{correlation} = \frac{\sum_{i=1}^{G}\sum_{j=1}^{G}(i \cdot j)p(i,j) - \mu_x\mu_y}{\sigma_x\sigma_y}$$

3. Difference entropy:
   Randomness of the difference of neighboring voxels' gray-levels

$$\text{Difference entropy} = -\sum_{k=0}^{G-1} p_{x-y}(k)\log(p_{x-y}(k))$$

4. Difference variance
   Variations of the difference of gray-levels between voxel-pairs

$$\text{Difference variance} = \sum_{k=0}^{G-1}(k - \mu_{x-y})^2 p_{x-y}(k)$$

5. Angular second moment (Energy):
   Image homogeneity

$$\text{Energy} = \sum_{i=1}^{G}\sum_{j=1}^{G}\{p(i,j)\}^2$$

6. Entropy:
   The measure of randomness of the gray-levels

$$\text{Entropy} = -\sum_{i=1}^{G}\sum_{j=1}^{G} p(i,j)\log(p(i,j))$$

7. Inverse difference moment:
   A measure of image homogeneity

$$\text{Inverse difference moment} = \sum_{i=1}^{G}\sum_{j=1}^{G}\frac{1}{1+|i-j|}p(i,j)$$

8. Information measure of correlation 1 (IMC1):
   Nonlinear gray-level dependence

$$\text{IMC1} = \frac{\text{HXY} - \text{HXY1}}{\max(\text{HX},\text{HY})}$$

9. Information measure of correlation 2(IMC2)

$$\text{IMC2} = (1 - e^{-2(\text{HXY2}-\text{HXY})})^{1/2}$$

10. Maximum correlation coefficient (MCC)

$$\text{MCC} = \sqrt{\text{second largest eigenvalue of B}}$$

where $B(i,j) = \sum_{k=1}^{G}\frac{p(i,k)p(j,k)}{p_x(i)p_y(j)}$

11. Sum average

$$\text{Sum average} = \sum_{k=2}^{2G} k p_{x+y}(k)$$

12. Sum entropy

$$\text{Sum entropy} = -\sum_{k=2}^{2G} p_{x+y}(k)\log(p_{x+y}(k))$$

13. Sum variance

$$\text{Sum variance} = \sum_{k=2}^{2G}(k - \sum_{k=2}^{2G} k p_{x+y}(k))^2 p_{x+y}(k)$$

14. Sum of squares (variance)

$$\text{Variance} = \sum_{i=1}^{G}\sum_{j=1}^{G}(i-\mu)^2\, p(i,j)$$

Group 5: Kinetic curve assessments:

Kinetic curve assessments characterize the physiological process of the uptake and washout of the contrast agent in a breast lesion during the dynamic imaging series and were extracted from the kinetic curve obtained from the most enhancing voxels within a lesion[5-6].

Two necessary notations are the following:
S_t: the signal intensity at time frame t, t=0 (i.e., pre-contrast frame), 1, 2, …, T−1 (i.e., the last post-contrast frame)
S^*: the maximum signal intensity, which is the maximum value of S_t over all time points.

1. Maximum enhancement (K1)

$$\text{Maximum enhancement} = (S^* - S_0)/S_0$$

2. Time to peak (K2)

   K2, time at which the maximum enhancement occurs

3. Uptake rate

$$\text{Uptake rate} = K1/K2$$

4. Washout rate

Washout rate

$$= \begin{cases} \dfrac{S^* - S_{T-1}}{S_0(\tau - K2)} & \text{if maximum enhancement is not at the last post-contrast frame, } \tau \text{ is the total imaging time} \\ 0 & \text{if maximum enhancement is at the last post-contrast frame} \end{cases}$$

5. Curve shape index

$$\text{Curve shape index} = \frac{S_{T-1} - S_1}{S_1 - S_0}$$

6. Enhancement at first post-contrast time point

$$\text{Enhancement at first post-contrast frame} = (S_1 - S_0)/S_0$$

7. Signal enhancement ratio

$$\text{Signal enhancement ratio} = (S_1 - S_0)/(S_{T-1} - S_0)$$

8. Volume of most enhancing voxels

   Number of voxels with the maximum enhancement

9. Total rate variation (TRV)

$$\mathrm{TRV} = \sum_{i=0}^{T-2} (v_{i+1} - v_i)^2$$

where $v_i = \frac{S_{i+1} - S_i}{\Delta t_i}$, Δt_i is the time elapsed between two frames

10. Normalized total rate variation (NTRV)

$$\mathrm{NTRV} = \frac{\mathrm{TRV}}{\left\{\max_{t=0,1,2,\ldots,T-1} S_t\right\}^2}$$

Group 6: Enhancement-variance kinetics

Enhancement-variance kinetics characterize the time course of the spatial variance of the enhancement within a breast tumor[6].

Some necessary notations are the following:
$S(r,t)$: signal intensity of a voxel with a spatial location r at time frame t, t=0, 1, 2 , …, $T-1$
$C(r,t) = \frac{S(r,t) - S(r,0)}{S(r,0)}$, contrast enhancement, t=0, 1, 2, …, $T-1$
L: the number of voxels within a lesion
$\overline{C}(t) = \frac{1}{L}\sum_{r=1}^{L} C(r,t)$, average contrast enhancement, t=0, 1, 2,…, $T-1$
$\mathrm{Var}(t) = \frac{1}{L-1}\sum_{r=1}^{L}\left[C(r,t) - \overline{C}(t)\right]^2$, spatial variance of the contrast enhancement within the lesion, $t = 0, 1, 2, \ldots, T-1$

1. Maximum variance of enhancement

$$\mathrm{Var}^* = \max_{t=0,1,2,\ldots,T-1} \mathrm{Var}(t)$$

2. Time to peak at maximum variance

   The time at which maximum variance of enhancement, i.e. Var^*, appears, denoted by Q.

3. Enhancement-variance increasing rate

$$\text{Enhancement-variance increasing rate} = \frac{\mathrm{Var}^*}{Q}$$

4. Enhancement-variance decreasing rate

   Enhancement-variance decreasing rate

$$= \begin{cases} \dfrac{\mathrm{Var}^* - \mathrm{Var}_{T-1}}{\tau - Q} & \text{if maximum variance is not at the last post-contrast frame, } \tau \text{ is total imaging time} \\ 0 & \text{if maximum variance is at the last post-contrast frame} \end{cases}$$

Section 2. Preparation of Genomic Data

All tumor cases were staged according to the American Joint Committee on Cancer (AJCC) staging system. The multi-layer genomic data of each case were generated by TCGA using a biospecimen from the primary tumor. The biospecimen was selected to be adjacent to the tissue block from which a diagnostic slide was obtained for the hospital to diagnose the patient. A pathology review was conducted on the tumor sample to determine whether it is qualified for making genomic data. A tumor sample was required to contain at least 60% tumor cell nuclei with less than 20% necrosis for inclusion in the study per TCGA protocol requirements.

We used TCGA-Assembler[8], a software package that automatically downloads, assembles, and processes TCGA data, to retrieve genomic data from the TCGA data server. Only samples with both genomic data and radiomic data were included in the analysis. We used the normalized read counts for RNA-seq data, which were generated by the Illumina HiSeq 2000 system and processed by the MapSplice genome alignment algorithm[9] and the RSEM gene expression estimation algorithm[10]. We used the RPM (Reads Per Million miRNAs mapped) values for miRNA-seq data. TCGA used the Affymetrix® Genome-Wide Human SNP Array 6.0 and the circular binary segmentation algorithm[11] to generate the copy numbers of DNA fragments. We used TCGA-Assembler to calculate an average copy number for each gene in each sample, which was the actual copy number value used in the analysis. TCGA somatic mutation data were generated using exome sequencing on the Illumina Genome Analyzer and HiSeq 2000 DNA sequencing platforms. Candidate mutations were identified using VarScan 2 for SNVs/Indels[12], SomaticSniper for SNVs[13], and GATK IndelGenotyper v2.0 for Indels[14]. The union of the mutation call sets from these callers were used and additional filtering and processing were taken by TCGA to ensure the quality of somatic mutation calls[15]. Note that TCGA used the Reverse Phase Protein Array (RPPA) to generate protein expression data, which measures 142 important proteins and phospho-proteins such as some transcription factors involved in cancer development.

Section 3. Overview of Radiogenomics Data

We conducted clustering analysis on tumor samples to provide an overview of the dataset and also associated the clustering results with the breast cancer clinical subtypes, such as different tumor pathological stages and molecular receptor status.

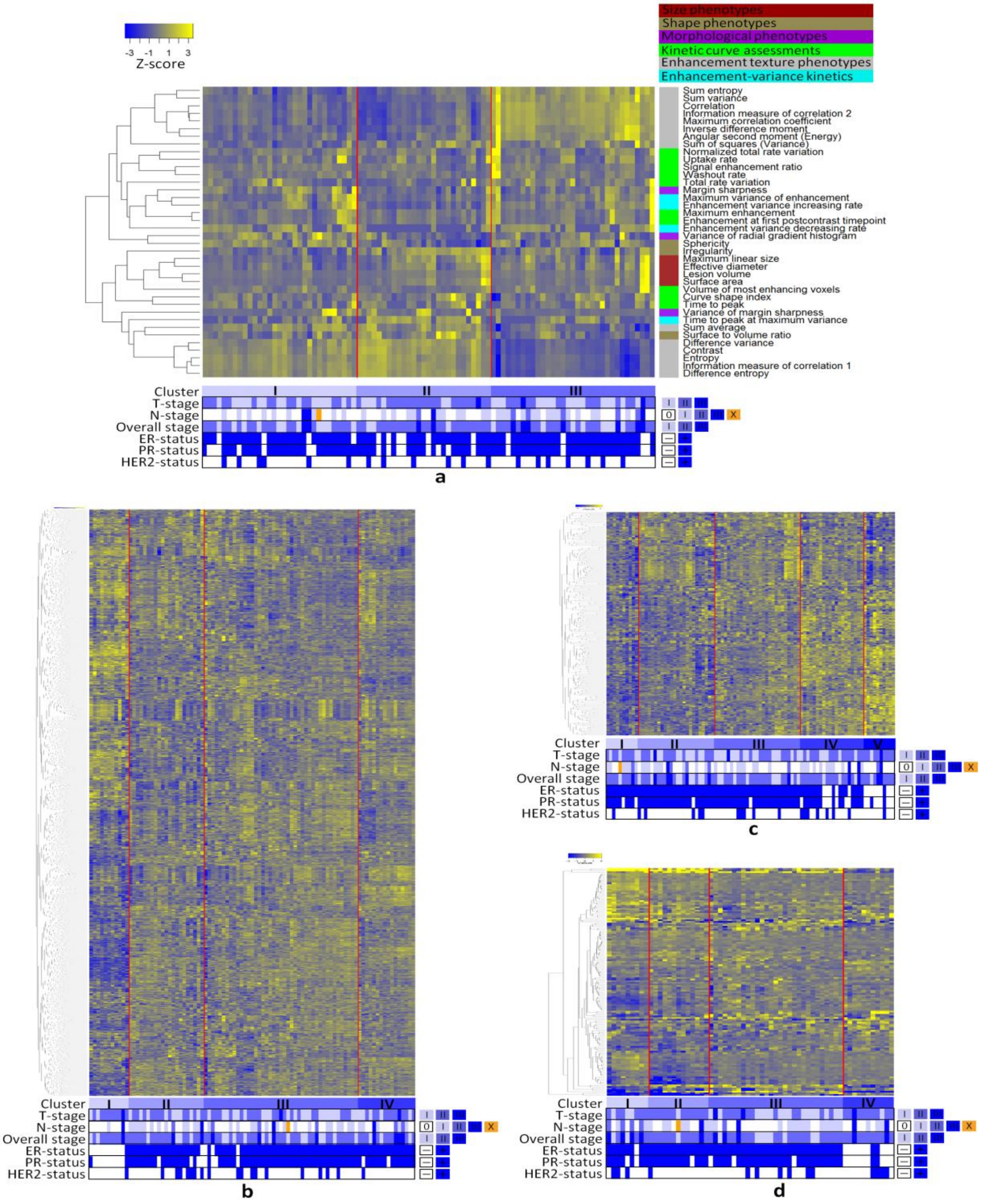

Figure S1 Clustering analysis of tumor samples based on different data platforms. In the heatmaps, the tumor samples (columns in the heatmaps) are grouped by the Affinity Propagation Clustering (APC) into clusters divided by red lines. All features (rows in the heatmaps) are organized by the hierarchical clustering based on the Euclidean distance and complete linkage function, for which a dendrogram is shown on the left. All features were transformed and standardized to obtain Z scores based on which the clustering and heatmap drawing were done. The pathological stages and the molecular receptor status of tumors are shown under the heatmaps. (a) Radiomic imaging data. (b) RNA-seq gene expression data. (c) MiRNA-seq data. (d) Protein expression data.

The radiomic data were preprocessed before clustering analysis. Values of three radiomic phenotypes, including *maximum variance of enhancement, enhancement-variance increasing rate*, and *enhancement-variance decreasing rate,* were log2 transformed to reduce the effects of extreme values. Zero values of *enhancement-variance decreasing rate* were replaced by the smallest positive value of the phenotype (over all patient samples) to allow the log2 transformation. All radiomic phenotypes were standardized to have a zero mean and a unit standard deviation for clustering analysis and producing heatmap. The Affinity Propagation Clustering (APC)[16] was applied to cluster the tumor samples and three tumor clusters with distinct radiomic profiles were identified, as shown in Fig. S1a. The genomic data were also preprocessed for clustering analysis and heatmap drawing. For RNA-seq data, gene filtering kept only genes with the CPM (Count Per Million reads mapped) ≥ 2 in at least a half of the samples, which resulted in 13177 genes for the analysis. 0 read counts in a sample were replaced by the minimum positive read count among all the genes in the sample. A Log2 transformation was then performed on the data. The expression values of each gene were standardized to have a zero mean and a unit standard deviation over all samples. The same procedure was used to preprocess the miRNA-seq data and 292 miRNAs were kept for the clustering analysis and heatmap drawing. Fig. S1b, c, and d present the tumor clustering results based on the gene expression data, miRNA expression data, and protein expression data, respectively.

Associations between the tumor clustering partitions and the classifications of tumors based on their pathological stage or receptor status were tested using the Fisher's exact test[17-18]. The obtained p-values were adjusted for multiple tests using the Benjamini–Hochberg (BH) procedure[19] and presented in Table S2. M-stage of the tumor samples was not considered for association analysis, because 89 out of the 91 samples are at M0 stage, indicating no clinical or radiographic evidence of distant metastases, and the rest two samples are at cM0(i+), indicating molecular or microscopical evidence of potential metastasis but no clinical or radiographic evidence.

The clustering partitions of tumors based on gene expressions, miRNA expressions, and protein expressions were found to be statistically significant in their associations with the status of Estrogen Receptor (ER) and Progesterone Receptor (PR), which means that ER+ patients and PR+ patients show different expression patterns from ER− patients and PR− patients, respectively, at multiple molecular levels. For the tumor clusters defined by mRNA expressions (Fig. S1b), most of the tumors in cluster II, III, and IV were ER+ and PR+, while almost all the tumors in cluster I were ER− and PR−. miRNA expressions defined five tumor clusters (Fig. S1c). Compared to clusters IV and V, clusters I, II, and III were dominated by ER+ and PR+ tumors. The four tumor clusters identified on protein expressions were statistically significantly associated with the tumor T stage with an adjusted p-value of 0.0171. Fig. S1d shows that clusters II and IV mainly consisted of T-II and T-III tumors and that clusters I, II, and III mainly included ER+ and PR+ tumors, while cluster IV was mainly composed of ER− and PR− tumors.

Table S2 The adjusted p-values from the Fisher's exact tests on the associations between tumor clustering partitions (defined by different data platforms) and tumor classifications by the pathological stage or molecular receptor status.

Pathological stage or receptor status	Radiomics	RNA-seq	miRNA-seq	Protein expression
**HER2 status**	1	0.284	0.593	0.378
**PR status**	0.325	7.49E-05	9.78E-06	0.000171
**ER status**	0.593	3.34E-08	8.26E-09	1.00E-07
**Overall stage**	0.144	0.726	0.325	0.122
**N stage**	0.284	0.284	0.072	0.122
**T stage**	0.122	0.325	0.192	0.0171

Section 4. Overview of Identified Statistically Significant Associations

Fig. 2b in the main paper summarizes the numbers of identified statistically significant associations between genomic features of different platforms and radiomic phenotypes of different categories. Associations were deemed as statistically significant if the adjusted p-value ≤ 0.05. Because a gene usually mutated in only very few patients (see Table S6 right), which resulted in a small number of samples with a mutation event and thus a relatively weak statistical power, we used less stringent criteria to call statistically significant associations of somatically mutated genes. The criteria are (1) p-value ≤ 0.05 and (2) the gene mutated in at least five patients. Based on the table in Fig. 2b, the Fisher's exact test[17-18] shows that the frequencies of statistically significant associations are dependent on the categories of genomic features and radiomic phenotypes (p-value $\leq 1.0\times10^{-8}$).

Table S3 shows that the identified associations are enriched for certain categories of genomic features and radiomic phenotypes, evaluated by the adjusted p-values from the Fisher's exact tests[17-18]. To explain how the enrichment significance was statistically evaluated, we take the associations between gene expressions of pathways and tumor size phenotypes as an example. The total number of genomic features is 186 (gene expressions of pathways) + 186 (copy number variations of pathways) + 292 (miRNA expressions) + 3734 (mutated genes) + 142 (protein expressions) = 4540, so there are 4540×38 = 172520 potential associations, among which 186×4 = 744 potential associations are between gene expressions of pathways and four tumor size phenotypes. From the table in Fig. 2b, 1404 statistically significant associations have been identified, among which 173 statistically significant associations are between gene expressions of pathways and size phenotypes. Based on these numbers, Fisher's exact test gives a p-value smaller than 1.0×10^{-30}, after correction over all 30 tests included in Table S3 using the BH procedure.

Table S3 The adjusted p-values resulted from the enrichment tests of statistically significant associations between each genomic platform and each radiomic phenotype category.

	Size phenotypes	Shape phenotypes	Morphological phenotypes	Kinetic curve assessments	Enhancement-variance kinetics	Enhancement texture phenotypes
**Transcriptional activities of pathways**	$<1.0\times10^{-30}$	$<1.0\times10^{-30}$	$<1.0\times10^{-30}$	$<1.0\times10^{-30}$	$<1.0\times10^{-30}$	$<1.0\times10^{-30}$
**Copy number variations of pathways**	7.95×10^{-8}	0.371	0.371	1	4.46×10^{-6}	1
**Mutated genes**	1	1	1	1	1	1
**miRNA expressions**	$<1.0\times10^{-30}$	1	1	1	1	0.000142
**Protein expressions**	0.0486	1	0.0244	1	1	0.904

A Chi-squared proportion test based on equal proportions of positive and negative associations was carried out for every combination of a genomic feature platform and a radiomic phenotype, when there are at least 10 statistically significant associations between them. The purpose was to examine whether a genomic feature platform is dominantly positively or negatively associated with a radiomic phenotype. The resulted p-values were then adjusted using the BH procedure over all the Chi-squared proportion tests.

Section 5. Associations between Genetic Pathways and Radiomic Phenotypes

We studied the associations between the transcriptional activities of genetic pathways documented by the Kyoto Encyclopedia of Genes and Genomes (KEGG) database[20] and the tumor radiomic phenotypes using the Gene Set Enrichment Analysis (GSEA). GSEA is based on known genetic pathways or functional gene modules. It studies the behavior of genes involved a pathway as a whole group in response to a biological condition. Various GSEA methods have been developed since it was first proposed in 2005[21]. However, there is no consolidated opinion regarding what methods should be preferred, because most methods have their own advantages and disadvantages[22]. Thus, Väremo et al. integrated multiple GSEA methods into a uniform workflow implemented as an R package called PIANO[22]. We used the PIANO package including nine different GSEA methods for our analysis. The nine methods were Stouffer's method, reporter features, tail strength, Wilcoxon rank-sum test, mean, median, sum, statistic, original GSEA, and Parametric Analysis of Gene set Enrichment (PAGE). They differ in the choice of gene-level statistic and the way of calculating gene-set-level statistic and evaluating its statistical significance. Refer to Väremo et al. (2013)[22] for a detailed description of these methods and the software package. The gene sets used in the analysis were KEGG pathways collected in the

Molecular Signature Database[21] that includes 186 genetic pathways or modules covering a wide range of genetic and molecular functionalities.

The RNA-seq data were filtered to remove the genes with unreliable expressions that could introduce significant noise or bias to the analysis results. A gene was excluded from the analysis, if its normalized read count was 0 in ten or more samples or its average read count over all samples was smaller than 8. After filtering, 15544 genes were kept.

For each of the 38 radiomic phenotypes, we performed the GSEA for identifying the KEGG pathways whose transcriptional changes associated with the change of radiomic phenotype. The gene-level statistics used to characterize the relationship between a gene's expression and a radiomic phenotype were the correlation coefficient and/or the p-value resulted from the Spearman rank correlation test[23], depending on the GSEA method. Stouffer's method, reporter features, and tail strength used p-values as the gene-level statistic and the signs of correlation coefficients were used to indicate the association direction. All other methods used correlation coefficient as the gene-level statistic. Nominal p-values for evaluating the statistical significance of association were calculated based on 30000 random gene sets. For each method, the False Discovery Rate (FDR) was controlled for each radiomic phenotype over its association tests with all KEGG pathways using the BH procedure[19]. Thus, we got nine adjusted p-values evaluating the association between each gene set and each radiomic phenotype, produced by nine different GSEA methods. An association was deemed as statistically significant, if the median of the nine adjusted p-values is no larger than 0.05, which actually required that more than a half of the methods simultaneously identified the association using a cutoff of 0.05 on the adjusted p-value. The associations between gene sets and radiomic phenotypes were tested separately for two different directions, i.e. positive association and negative association.

Table S4 The median adjusted p-values resulted from the association tests between all radiomic phenotypes and the transcriptional activities of all KEGG pathways. The results are summarized in two different directions, i.e. positive association and negative association.
[Table S4 is in the excel file]

Table S4 provides the median adjusted p-values for all association tests between pathway transcriptional activities and radiomic phenotypes. Totally, 1103 statistically significant associations were identified. Fig. 3 in the main text shows some of the associations that involve cancer-related pathways. Besides the findings elaborated in the main text, some other findings are the following.

P53 Signaling Pathway The P53 signaling pathway takes a tumor suppressive role by causing cell cycle arrest, cellular senescence, and apoptosis in normal condition. Its transcriptional activity is positively associated with the tumor size and tumor shape irregularity (Fig. 3 in the main text), which indicates the activation of this pathway during tumor development. It also has a positive association with the radiomic phenotype *maximum enhancement*, indicating that tumors with an active P53 signaling pathway tend to have increased permeability of blood vessels.

MAPK Signaling Pathway The MAPK signaling pathway is critical for cell proliferation and migration involved in cancer development. We see a statistically significant negative association between the MAPK activity and *time to peak at maximum variance* (Fig. 3 in the main text), which suggests that tumors with a strong MAPK pathway activity tend to quickly reach their maximum value of enhancement variation, which measures the heterogeneity of blood uptake within a tumor.

DNA Repair Pathways Two related DNA repair pathways, the mismatch repair pathway and the base excision repair pathway, play key roles in maintaining genomic stability and preventing DNA mutations from becoming permanent in dividing cells. We found that the pathways' transcriptional activities were positively correlated with all tumor size phenotypes (Fig. 3 in main text), indicating their activity during tumor progression trying to prevent DNA mutations. The mismatch repair pathway is also positively correlated with leaky microvessels characterized by *maximum enhancement* and *enhancement at the first post-contrast time point*.

Besides gene expressions, we also used the same GSEA scheme to identify the KEGG pathways whose gene copy number variations statistically significantly associated with the radiomic phenotypes. 15000 genes whose DNA copy number varies most (measured by the standard deviation over all patients) were used for analysis. Gene sets with a median adjusted p-value ≤ 0.05 over all nine methods were deemed as statistically significant. Fig. S2 shows all the statistically significant associations between radiomic phenotypes and copy number variations of KEGG pathways.

We take the median of nine adjusted p-values obtained by nine different GSEA methods and then apply the cutoff of 0.05 on the median adjusted p-values to call significant associations. We do so because of the following two reasons.

(1) The nine methods differ in the choice of gene-level statistic and the ways of calculating gene-set-level statistic and evaluating its statistical significance[22]. Unfortunately, there is no consolidated opinion regarding what methods should be preferred, because most methods have their own advantages, disadvantages, and statistical assumptions[22]. So whether a method is suitable/best for statistical inference on a particular dataset is data-dependent. It might not be the best to assume all methods are suitable for a given data set. Making a significance call base on the median of adjusted p-values from the nine methods is basically a majority vote. If more than a half of the methods (i.e. five methods) vote for a significant call, the association is reported.

(2) Requiring all nine methods to agree on the significance call is more stringent and can reduce false discoveries, but may also increase false non-discoveries. To show this, we tried requiring that all nine methods must reach the statistical significance cutoff (adjusted p-value = 0.05) in order to call a significant association. As a result, the number of significant associations involving pathway transcriptional activities decreased from 1103 to 503, and the number of significant associations involving pathway copy number variations decreased from 88 to 26.

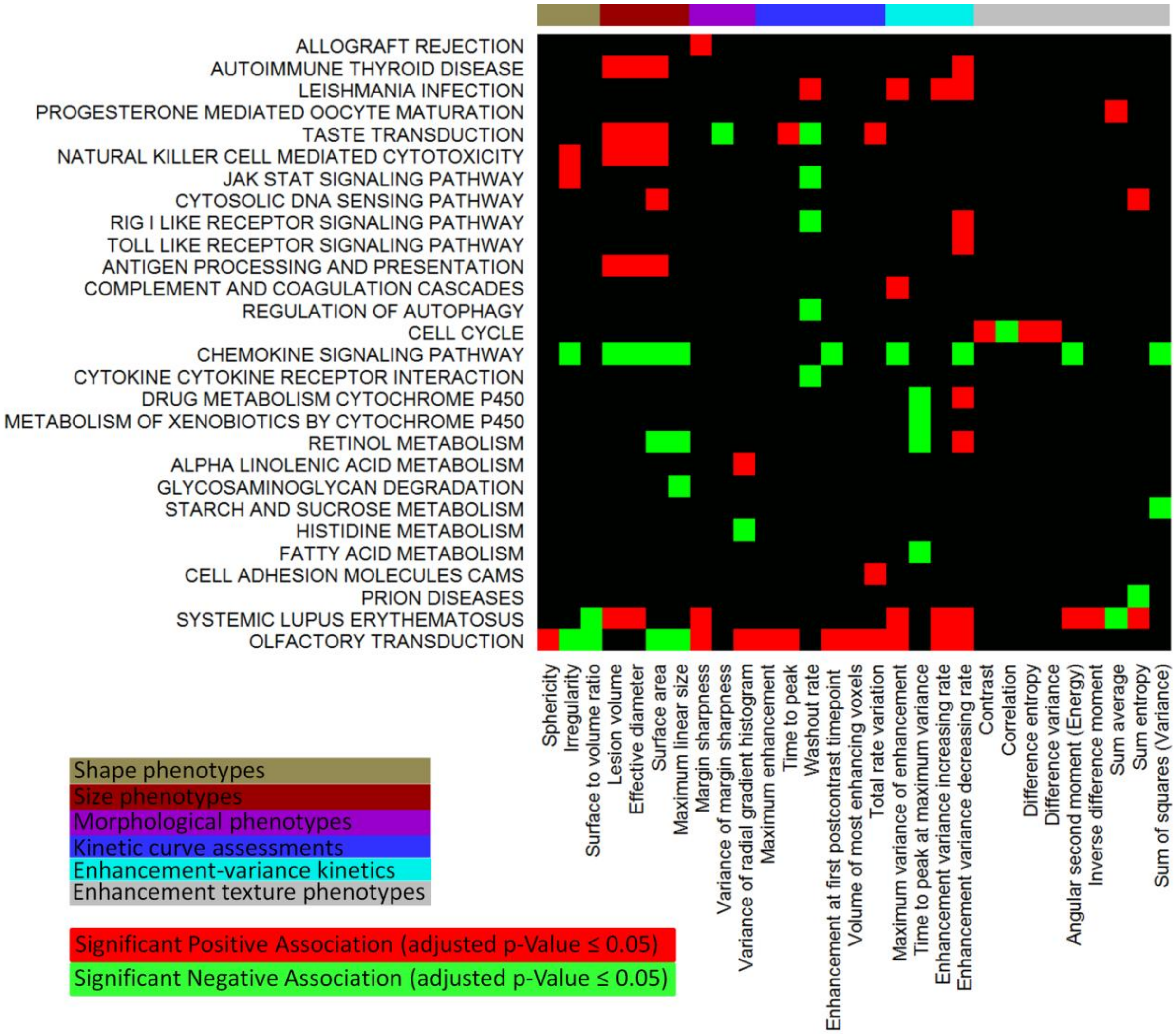

Figure S2 A heatmap presentation of all statistically significant associations between radiomic phenotypes and copy number variations of KEGG pathways. In the heatmap, genetic pathways are rows and radiomic phenotypes are columns.

Section 6. Associations between miRNA Expressions and Radiomic Phenotypes

The radiomic data were preprocessed before carrying out the analysis. Values of three radiomic phenotypes, including *maximum variance of enhancement*, *enhancement-variance increasing rate*, and *enhancement-variance decreasing rate*, were log2 transformed to reduce the effect of extreme values. 0 values of *enhancement-variance decreasing rate* were replaced by the smallest positive value of the phenotype (over all patient samples) to allow the log2 transformation. Gene filtering was performed to keep the miRNAs whose RPM values are no less than 2 in at least a

half of the samples, which resulted in 292 miRNAs for the analysis. 0 RPMs in a sample were replaced by the minimum positive RPM among all miRNAs in the sample. A log2 transformation of the data was then performed.

For each miRNA and each radiomic phenotype, a linear regression with adjustments of the patient age and the tumor grade was used to fit the values of the radiomic phenotype and to examine whether the miRNA expression has a statistically significant effect on the phenotype, which was formulated as

$$x_i = \beta_0 + \beta_1 m_i + \beta_2 a_i + \beta_3 g_{2,i} + \beta_4 g_{3,i} + \varepsilon, \quad (1)$$

where x_i was the value of the radiomic phenotype for patient i, m_i was the expression level of the miRNA in patient i, a_i was the age of patient i, $g_{2,i}$ and $g_{3,i}$ were two 0/1 indicators coding the patient tumor stage (stage I: $g_{2,i} = 0$ and $g_{3,i} = 0$; stage II: $g_{2,i} = 1$ and $g_{3,i} = 0$; stage III: $g_{2,i} = 0$ and $g_{3,i} = 1$). A p-value evaluating the statistical significance of β_1 was calculated and adjusted over the tests of all miRNAs with the particular radiomic phenotype. We collected a list of oncogenic and tumor-suppressive miRNAs by literature survey[24-27]. Fig. 4a in the main text shows the obtained statistically significant (adjusted p-value $\leq$ 0.05) associations between the cancer-related miRNAs and the radiomic phenotypes. Table S5 summarizes all the statistically significant associations between miRNA expressions and radiomic phenotypes.

Table S5 Analysis results of the statistically significant associations between radiomic phenotypes and miRNA expressions.

miRNA symbol	Radiomic phenotype	Adjusted p-value	Regression coefficient
hsa-let-7b	Entropy	1.97E-02	-3.86E-02
hsa-let-7b	Difference variance	3.45E-02	-1.47E+00
hsa-let-7b	Contrast	4.34E-02	-3.17E+00
hsa-let-7b	Difference entropy	4.45E-02	-4.61E-02
hsa-let-7b	Angular second moment (Energy)	4.49E-02	8.98E-05
hsa-let-7b	Correlation	4.66E-02	2.21E-02
hsa-let-7b	Maximum correlation coefficient	4.84E-02	2.01E-02
hsa-let-7b	Information measure of correlation 1	4.85E-02	-1.17E-02
hsa-let-7d	Lesion volume	3.02E-02	2.89E+03
hsa-let-7d	Effective diameter	4.79E-02	1.88E+00
hsa-mir-106a	Effective diameter	4.48E-02	1.91E+00

hsa-mir-106b	Lesion volume	1.50E-03	4.14E+03
hsa-mir-106b	Effective diameter	6.71E-03	2.53E+00
hsa-mir-106b	Surface area	2.04E-02	2.09E+03
hsa-mir-10b	Effective diameter	3.70E-02	-1.94E+00
hsa-mir-128-1	Lesion volume	1.10E-02	3.36E+03
hsa-mir-128-1	Effective diameter	2.25E-02	2.08E+00
hsa-mir-128-2	Lesion volume	1.10E-02	3.36E+03
hsa-mir-128-2	Effective diameter	2.15E-02	2.10E+00
hsa-mir-1296	Entropy	3.63E-02	3.63E-02
hsa-mir-1296	Angular second moment (Energy)	4.59E-02	-9.03E-05
hsa-mir-1301	Lesion volume	2.16E-02	3.09E+03
hsa-mir-1301	Effective diameter	2.25E-02	2.11E+00
hsa-mir-1307	Effective diameter	1.52E-02	2.27E+00
hsa-mir-1307	Lesion volume	4.41E-02	2.75E+03
hsa-mir-130b	Lesion volume	1.07E-02	3.57E+03
hsa-mir-130b	Effective diameter	1.52E-02	2.28E+00
hsa-mir-130b	Surface area	4.74E-02	1.82E+03
hsa-mir-144	Lesion volume	5.34E-03	3.70E+03
hsa-mir-144	Surface area	7.25E-03	2.27E+03
hsa-mir-1468	Lesion volume	2.30E-02	3.06E+03
hsa-mir-17	Difference variance	4.12E-02	1.44E+00
hsa-mir-185	Lesion volume	3.03E-03	4.02E+03
hsa-mir-185	Effective diameter	6.71E-03	2.54E+00
hsa-mir-185	Surface area	7.25E-03	2.31E+03
hsa-mir-18a	Lesion volume	2.46E-02	2.96E+03
hsa-mir-18a	Difference variance	4.28E-02	1.39E+00

hsa-mir-190b	Effective diameter	4.79E-02	-1.86E+00
hsa-mir-19a	Difference variance	2.74E-02	1.56E+00
hsa-mir-19a	Entropy	3.63E-02	3.55E-02
hsa-mir-19a	Contrast	4.34E-02	3.19E+00
hsa-mir-19a	Correlation	4.66E-02	-2.16E-02
hsa-mir-19a	Maximum correlation coefficient	4.84E-02	-2.04E-02
hsa-mir-19b-2	Difference variance	1.21E-02	1.79E+00
hsa-mir-19b-2	Entropy	1.57E-02	4.27E-02
hsa-mir-19b-2	Angular second moment (Energy)	2.35E-02	-1.02E-04
hsa-mir-19b-2	Correlation	2.67E-02	-2.54E-02
hsa-mir-19b-2	Maximum correlation coefficient	2.78E-02	-2.42E-02
hsa-mir-19b-2	Contrast	2.98E-02	3.72E+00
hsa-mir-19b-2	Information measure of correlation 1	3.60E-02	1.30E-02
hsa-mir-19b-2	Information measure of correlation 2	3.64E-02	-2.08E-02
hsa-mir-19b-2	Difference entropy	4.29E-02	5.16E-02
hsa-mir-20a	Difference variance	4.14E-02	1.41E+00
hsa-mir-2355	Entropy	1.57E-02	4.11E-02
hsa-mir-2355	Difference variance	2.74E-02	1.57E+00
hsa-mir-2355	Angular second moment (Energy)	3.47E-02	-9.48E-05
hsa-mir-2355	Contrast	4.34E-02	3.18E+00
hsa-mir-2355	Difference entropy	4.37E-02	4.71E-02
hsa-mir-2355	Correlation	4.66E-02	-2.12E-02
hsa-mir-2355	Maximum correlation coefficient	4.84E-02	-2.00E-02
hsa-mir-25	Lesion volume	4.27E-05	4.81E+03
hsa-mir-25	Surface area	9.65E-04	2.60E+03
hsa-mir-25	Effective diameter	4.83E-03	2.72E+00

hsa-mir-25	Volume of most enhancing voxels	1.39E-02	4.16E+01
hsa-mir-28	Lesion volume	1.10E-02	3.45E+03
hsa-mir-28	Effective diameter	2.15E-02	2.15E+00
hsa-mir-28	Surface area	4.74E-02	1.80E+03
hsa-mir-299	Difference variance	3.90E-02	-1.43E+00
hsa-mir-30a	Difference variance	3.45E-02	-1.55E+00
hsa-mir-320b-2	Effective diameter	3.11E-02	2.04E+00
hsa-mir-324	Lesion volume	1.10E-02	3.40E+03
hsa-mir-324	Effective diameter	2.30E-02	2.06E+00
hsa-mir-328	Lesion volume	3.81E-02	2.78E+03
hsa-mir-330	Effective diameter	6.71E-03	2.48E+00
hsa-mir-330	Lesion volume	2.30E-02	3.04E+03
hsa-mir-339	Entropy	2.93E-02	3.75E-02
hsa-mir-339	Difference variance	3.45E-02	1.50E+00
hsa-mir-339	Angular second moment (Energy)	4.49E-02	-9.09E-05
hsa-mir-339	Correlation	4.66E-02	-2.11E-02
hsa-mir-339	Maximum correlation coefficient	4.84E-02	-2.01E-02
hsa-mir-33a	Lesion volume	3.81E-02	2.82E+03
hsa-mir-345	Lesion volume	1.17E-02	3.34E+03
hsa-mir-34a	Entropy	3.63E-02	3.60E-02
hsa-mir-34a	Difference variance	3.90E-02	1.44E+00
hsa-mir-34a	Correlation	4.66E-02	-2.09E-02
hsa-mir-34a	Maximum correlation coefficient	4.84E-02	-2.08E-02
hsa-mir-362	Difference variance	1.21E-02	1.75E+00
hsa-mir-362	Entropy	1.65E-02	4.01E-02
hsa-mir-362	Contrast	3.28E-02	3.52E+00

hsa-mir-362	Correlation	3.47E-02	-2.38E-02
hsa-mir-362	Difference entropy	4.37E-02	4.77E-02
hsa-mir-362	Maximum correlation coefficient	4.84E-02	-2.13E-02
hsa-mir-362	Information measure of correlation 1	4.85E-02	1.18E-02
hsa-mir-421	Effective diameter	4.91E-03	2.65E+00
hsa-mir-421	Lesion volume	1.19E-02	3.28E+03
hsa-mir-421	Surface area	3.39E-02	1.92E+03
hsa-mir-451	Lesion volume	1.14E-02	3.36E+03
hsa-mir-451	Surface area	2.29E-02	2.02E+03
hsa-mir-486	Lesion volume	2.30E-02	3.10E+03
hsa-mir-486	Surface area	3.39E-02	1.94E+03
hsa-mir-505	Lesion volume	1.19E-02	3.30E+03
hsa-mir-505	Effective diameter	1.52E-02	2.24E+00
hsa-mir-576	Lesion volume	2.30E-02	3.11E+03
hsa-mir-576	Effective diameter	4.48E-02	1.97E+00
hsa-mir-584	Lesion volume	1.91E-02	3.15E+03
hsa-mir-584	Effective diameter	3.49E-02	1.98E+00
hsa-mir-584	Surface area	4.40E-02	1.84E+03
hsa-mir-589	Lesion volume	1.94E-02	3.15E+03
hsa-mir-589	Surface area	3.39E-02	1.92E+03
hsa-mir-590	Lesion volume	1.24E-02	3.28E+03
hsa-mir-590	Entropy	1.57E-02	4.11E-02
hsa-mir-590	Angular second moment (Energy)	2.35E-02	-1.05E-04
hsa-mir-590	Difference variance	2.40E-02	1.64E+00
hsa-mir-590	Effective diameter	3.49E-02	2.00E+00
hsa-mir-590	Contrast	4.34E-02	3.21E+00

hsa-mir-590	Correlation	4.66E-02	-2.16E-02
hsa-mir-590	Maximum correlation coefficient	4.84E-02	-2.13E-02
hsa-mir-652	Effective diameter	1.66E-02	2.26E+00
hsa-mir-652	Lesion volume	2.46E-02	3.03E+03
hsa-mir-744	Lesion volume	2.46E-02	2.98E+03
hsa-mir-769	Lesion volume	4.14E-03	3.83E+03
hsa-mir-769	Effective diameter	9.10E-03	2.40E+00
hsa-mir-769	Surface area	2.21E-02	2.06E+03
hsa-mir-93	Lesion volume	1.19E-02	3.28E+03
hsa-mir-93	Effective diameter	1.91E-02	2.15E+00
hsa-mir-93	Surface area	4.74E-02	1.76E+03
hsa-mir-937	Effective diameter	1.52E-02	2.21E+00
hsa-mir-937	Lesion volume	4.40E-02	2.72E+03
hsa-mir-940	Lesion volume	2.73E-02	2.96E+03
hsa-mir-98	Lesion volume	4.14E-03	3.78E+03
hsa-mir-98	Effective diameter	9.10E-03	2.40E+00
hsa-mir-98	Surface area	2.63E-02	1.98E+03

Section 7. Associations between Protein Expressions and Radiomic Phenotypes

The radiomic data were preprocessed as described in the previous section. The RPPA data included the expression levels of 142 proteins and phospho-proteins related to cancer. We used the level-3 data from TCGA, which were log2 transformed. A linear regression model was used to study the association between each radiomic phenotype and the expression of each protein, following equation (1), where m_i here denoted the protein's expression level in patient i. A p-value evaluating the statistical significance of β_1 was calculated and adjusted among the tests of all proteins with the radiomic phenotype. Fig. 4b in the main text shows all the identified statistically significant (adjusted p-value ≤ 0.05) associations. Some interesting findings have

been observed. For example, MEK1 is a protein kinase encoded by gene MAP2K1 that activates the extracellular signal-regulated kinases (ERKs) in the MAPK signaling pathway. The expression of MEK1 is statistically significantly negatively associated with *lesion volume*.

Section 8. Associations between Somatic Gene Mutations and Radiomic Phenotypes

The radiomic data were preprocessed as described in Section 6. For each mutation and each radiomic phenotype, a linear regression was used to fit the value of the radiomic phenotype and examine whether the mutation has a significant effect on the phenotype, following equation (1), where m_i here was a 0/1 indicator indicating whether patient i has the mutation.

Since the number of patients with exactly the same mutation was quite small (see Table S6 left) and might not provide reliable statistics, we only studied the associations of radiomic phenotypes with the most frequent mutation, which was A/A > A/G in PIK3CA at chromosome 3 and position 178952085 that occurred in 10 patients. P-values were calculated to evaluate the statistical significance of β_1 and were adjusted by the BH procedure to control FDR over the 38 tests. No statistically significant association was found. The smallest p-value and adjusted p-value obtained in the analysis were 0.148 and 0.820, respectively, for the association between *uptake rate* and the mutation.

Table S6 The numbers of somatic mutations (left) and somatically mutated genes (right) with different occurrence frequencies among patients.

Somatic mutations		Somatically mutated genes	
**Number of patients with the same mutation**	**Number of mutations**	**Number of patients with the same gene mutated**	**Number of genes**
10	1	31	1
4	3	29	1
3	1	13	1
2	17	12	1
1	4732	7	2
**Total**	**4754**	6	3
		5	6
		4	24
		3	82
		2	507
		1	3106
		**Total**	**3734**

Alternatively, we examined whether a gene underwent mutation was associated with a significant change in radiomic tumor phenotypes, regardless what mutations occurred to the gene and where they occurred in the gene. In this way, the patient group with somatic mutations (Table S6 right) can be larger than considering the exact same mutation occurring among patients (Table S6 left). The linear regression analysis was applied for every pair of mutated gene and radiomic phenotype according to equation (1), with m_i now being a 0/1 variable indicating whether patient i has at least one somatic mutation in the gene. The BH procedure was taken to control the FDR over the association tests between a radiomic phenotype and all mutated genes. Due to the weak statistic power associated with the usually small number of patients with the mutation events, we used loose criteria to call statistically significant associations, which were p-value ≤ 0.05 and that the gene mutated in at least five patients. Table S7 summarizes all the identified associations. Some of the associations do not have statistically significant adjusted p-values (<= 0.05) after the correction for multiple tests, although non-significant adjusted p-values do not guarantee that the specific associations are untrue.

Table S7 The analysis results of associations between somatically mutated genes and radiomic phenotypes. Only the associations in which a gene mutated in at least five patients and the p-value ≤ 0.05 are included.

Gene symbol	Radiomic phenotype	p-Value	Adjusted p-value	Regression coefficient	Number of samples with mutation in the gene	Number of samples without mutation in the gene
PIK3CA	Maximum correlation coefficient	2.16E-02	6.00E-01	3.10E-02	29	62
PIK3CA	Uptake rate	2.76E-02	5.93E-01	-6.51E-03	29	62
PIK3CA	Angular second moment (Energy)	3.86E-02	9.64E-01	1.21E-04	29	62
PIK3CA	Information measure of correlation 2	4.09E-02	7.70E-01	2.41E-02	29	62
TTN	Time to peak	1.23E-02	5.38E-02	6.99E+01	13	78
TTN	Curve shape index	3.27E-02	3.64E-01	1.08E-01	13	78
GATA3	Irregularity	5.31E-03	5.37E-01	-9.12E-02	12	79
GATA3	Effective diameter	7.82E-03	6.64E-01	-5.31E+00	12	79
GATA3	Maximum linear size	2.02E-02	8.97E-01	-1.03E+01	12	79
GATA3	Uptake rate	3.51E-02	5.93E-01	8.89E-03	12	79
GATA3	Surface area	3.59E-02	9.99E-01	-3.77E+03	12	79
GATA3	Sum entropy	4.01E-02	6.34E-01	-7.43E-03	12	79
GATA3	Time to peak	4.92E-02	1.98E-01	-5.72E+01	12	79
CROCCP2	Total rate variation	1.35E-03	9.03E-02	2.08E+02	6	85
CROCCP2	Maximum enhancement	2.75E-03	2.71E-01	1.07E+00	6	85
CROCCP2	Enhancement at first postcontrast timepoint	4.15E-03	4.19E-01	1.00E+00	6	85
CROCCP2	Signal enhancement ratio	5.55E-03	9.95E-01	4.20E-01	6	85
CROCCP2	Washout rate	2.58E-02	9.92E-01	1.11E-03	6	85
MACF1	Time to peak	8.11E-04	4.10E-03	1.25E+02	6	85

NEB	Total rate variation	1.77E-03	1.16E-01	2.06E+02	6	85
NEB	Maximum enhancement	2.24E-03	2.26E-01	1.10E+00	6	85
NEB	Enhancement at first postcontrast timepoint	4.70E-03	4.61E-01	1.00E+00	6	85
NEB	Angular second moment (Energy)	5.67E-03	5.73E-01	3.04E-04	6	85
NEB	Normalized total rate variation	1.04E-02	9.73E-01	5.47E-05	6	85
NEB	Enhancement variance increasing rate	1.21E-02	8.25E-01	2.54E+00	6	85
NEB	Maximum variance of enhancement	1.29E-02	6.20E-01	2.36E+00	6	85
NEB	Information measure of correlation 1	1.64E-02	7.97E-01	-3.35E-02	6	85
NEB	Entropy	1.80E-02	8.14E-01	-1.06E-01	6	85
NEB	Inverse difference moment	2.26E-02	9.32E-01	2.47E-02	6	85
NEB	Sum variance	2.81E-02	6.99E-01	1.12E+01	6	85
NEB	Difference entropy	3.26E-02	7.26E-01	-1.20E-01	6	85
NEB	Information measure of correlation 2	3.99E-02	7.70E-01	4.60E-02	6	85
NEB	Maximum correlation coefficient	4.59E-02	6.00E-01	5.12E-02	6	85
NEB	Volume of most enhancing voxels	4.88E-02	9.86E-01	8.51E+01	6	85
MAP2K4	Time to peak	4.98E-03	2.41E-02	1.16E+02	5	86
MAP2K4	Uptake rate	2.27E-02	5.93E-01	-1.38E-02	5	86
MUC16	Time to peak at maximum variance	7.94E-03	1.80E-01	-1.61E+02	5	86
SYNE1	Time to peak	1.94E-04	2.49E-03	1.51E+02	5	86
SYNE1	Volume of most enhancing voxels	1.61E-02	9.86E-01	1.12E+02	5	86
SYNE1	Uptake rate	4.51E-02	5.93E-01	-1.22E-02	5	86
USH2A	Sum entropy	2.87E-02	6.34E-01	1.13E-02	5	86
USH2A	Sum average	3.15E-02	2.47E-01	-5.94E-01	5	86
USH2A	Time to peak	4.23E-02	1.71E-01	8.43E+01	5	86
USH2A	Inverse difference moment	4.32E-02	9.32E-01	2.37E-02	5	86
ZNF540	Variance of margin sharpness	4.25E-02	5.71E-01	4.50E-04	5	86

We also studied the associations between radiomic phenotypes and somatic gene mutations at the pathway level, by comparing the measurements of radiomic phenotypes for patients with gene mutations in a KEGG pathway versus those without any mutation. A linear regression analysis was applied for each pair of KEGG pathway and radiomic phenotype according to equation (1), where m_i here is a 0/1 variable indicating whether patient i has at least one somatic mutation in the genes of the pathway. The BH procedure was taken to control the FDR for the association tests between a radiomic phenotype and all KEGG pathways. Table S8 shows all the associations in which the pathway has gene mutations in at least five patients and the obtained adjusted p-value ≤ 0.05.

Table S8 The analysis results of associations between radiomic phenotypes and somatic gene mutations at the pathway level. Only the associations in which the pathway has mutated genes in at least five patients and the adjusted p-value ≤ 0.05 are included in the table.

Pathway	Radiomic phenotype	Adjusted p-value	Regression coefficient	Number of samples with mutation in the pathway	Number of samples without mutation in the pathway
PYRIMIDINE METABOLISM	Time to peak	0.0226	82.31	14	77
PHENYLALANINE METABOLISM	Time to peak	0.0160	144.32	5	86
SELENOAMINO ACID METABOLISM	Time to peak	0.0458	93.26	8	83
GLUTATHIONE METABOLISM	Time to peak	0.0005	185.24	5	86
STARCH AND SUCROSE METABOLISM	Time to peak	0.0458	82.43	10	81
INOSITOL PHOSPHATE METABOLISM	Time to peak	0.0162	62.74	43	48
GLYCOSYLPHOSPHATIDYLINOSITOL GPI ANCHOR BIOSYNTHESIS	Time to peak	0.0104	154.38	5	86
RETINOL METABOLISM	Time to peak	0.0370	90.67	9	82
TERPENOID BACKBONE BIOSYNTHESIS	Time to peak	0.0171	132.05	5	86
METABOLISM OF XENOBIOTICS BY CYTOCHROME P450	Time to peak	0.0458	85.37	9	82
DRUG METABOLISM CYTOCHROME P450	Time to peak	0.0171	107.76	8	83
RNA POLYMERASE	Time to peak	0.0171	104.64	9	82
BASAL TRANSCRIPTION FACTORS	Time to peak	0.0320	111.23	6	85
DNA REPLICATION	Time to peak	0.0226	97.95	9	82
BASE EXCISION REPAIR	Time to peak	0.0162	131.40	6	85
NUCLEOTIDE EXCISION REPAIR	Time to peak	0.0320	86.70	10	81
MISMATCH REPAIR	Time to peak	0.0458	103.25	6	85
PHOSPHATIDYLINOSITOL SIGNALING SYSTEM	Time to peak	0.0171	61.52	46	45
P53 SIGNALING PATHWAY	Effective diameter	0.0326	4.78	42	49
LYSOSOME	Time to peak	0.0104	106.02	12	79
PEROXISOME	Time to peak	0.0370	89.82	9	82
TYPE I DIABETES MELLITUS	Time to peak	0.0150	133.48	6	85
VIBRIO CHOLERAE INFECTION	Time to peak	0.0296	82.39	12	79
EPITHELIAL CELL SIGNALING IN HELICOBACTER PYLORI INFECTION	Time to peak	0.0162	90.31	13	78
ASTHMA	Time to peak	0.0259	129.36	5	86
ALLOGRAFT REJECTION	Time to peak	0.0259	129.36	5	86
GRAFT VERSUS HOST DISEASE	Time to peak	0.0320	121.15	5	86

Section 9. Associations between Non-BRCA Cancer Pathways and BRCA Radiomic Phenotypes

Table S9 summarizes the numbers of statistically significant (adjusted p-value ≤ 0.05) associations between the radiomic phenotypes of Invasive Breast Carcinoma (BRCA) and the transcriptional activities of KEGG pathways dedicated to cancer types other than BRCA. The basal cell carcinoma has the highest number of associations, followed by the small cell lung cancer and glioma.

Table S9 The numbers of statistically significant associations between the non-BRCA KEGG cancer pathways and the BRCA radiomic phenotypes

Non-BRCA Cancer Pathway	Number of Associated BRCA Radiomic Phenotypes
Pancreatic cancer	1
Glioma	5
Thyroid cancer	0
Acute myeloid leukemia	2
Chronic myeloid leukemia	0
Basal cell carcinoma	12
Melanoma	0
Renal cell carcinoma	1
Bladder cancer	2
Prostate cancer	0
Endometrial cancer	1
Small cell lung cancer	5
Non small cell lung cancer	4

References

1 Chen, W., Giger, M. L. & Bick, U. A fuzzy c-means (FCM)-based approach for computerized segmentation of breast lesions in dynamic contrast-enhanced MR images. *Acad. Radiol.* **13**, 63-72 (2006).

2 Gilhuijs, K. G. A., Giger, M. L. & Bick, U. Automated analysis of breast lesions in three dimensions using dynamic magnetic resonance imaging. *Med. Phys.* **25**, 1647-1654 (1998).

3 Haralick, R. M., Shanmugam, K. & Dinstein, I. Textural features for image classification. *IEEE Trans. Syst. Man Cybern.* **6**, 610-621 (1973).

4 Chen, W., Giger, M. L., Li, H., Bick, U. & Newstead, G. M. Volumetric texture analysis of breast lesions on contrast-enhanced magnetic resonance images. *Magn. Reson. Med.* **58**, 562-571 (2007).

5 Chen, W., Giger, M. L., Bick, U. & Newstead, G. M. Automatic identification and classification of characteristic kinetic curves of breast lesions on DCE-MRI. *Med. Phys.* **33**, 2878-2887 (2006).
6 Chen, W., Giger, M. L., Lan, L. & Bick, U. Computerized interpretation of breast MRI: investigation of enhancement-variance dynamics. *Med. Phys.* **31**, 1076-1082 (2004).
7 Chen, W. *et al.* Computerized assessment of breast lesion malignancy using DCE-MRI robustness study on two independent clinical datasets from two manufacturers. *Acad. Radiol.* **17**, 822-829, doi:doi: 10.1016/j.acra.2010.03.007 (2010).
8 Zhu, Y., Qiu, P. & Ji, Y. TCGA-Assembler: open-source software for retrieving and processing TCGA data. *Nat. Methods* **11**, 599-600 (2014).
9 Wang, K. *et al.* MapSplice: Accurate mapping of RNA-seq reads for splice junction discovery. *Nucleic Acids Res.* **38**, e178, doi:10.1093/nar/gkq622 (2010).
10 Li, B. & Dewey, C. N. RSEM: accurate transcript quantification from RNA-Seq data with or without a reference genome. *BMC Bioinformatics* **12**, doi:10.1186/1471-2105-12-323 (2011).
11 Olshen, A. B., Venkatraman, E. S., Lucito, R. & Wigler, M. Circular binary segmentation for the analysis of array-based DNA copy number data. *Biostatistics* **5**, 557-572 (2004).
12 Koboldt, D. C. *et al.* VarScan 2: somatic mutation and copy number alteration discovery in cancer by exome sequencing. *Genome Res.* **22**, 568-576, doi:doi: 10.1101/gr.129684.111 (2012).
13 Larson, D. E. *et al.* SomaticSniper: identification of somatic point mutations in whole genome sequencing data. *Bioinformatics* **28**, 311-317 (2012).
14 DePristo, M. *et al.* A framework for variation discovery and genotyping using next-generation DNA sequencing data. *Nature Genet.* **43**, 491-498 (2011).
15 Network, T. C. G. A. Comprehensive molecular portraits of human breast tumours. *Nature* **490**, 61-70, doi:10.1038/nature11412 (2012).
16 Frey, B. J. & Dueck, D. Clustering by Passing Messages Between Data Points. *Science* **315**, 972–976 (2007).
17 Mehta, C. R. & Patel, N. R. Algorithm 643. FEXACT: A Fortran subroutine for Fisher's exact test on unordered r*c contingency tables. *ACM Trans. Math. Softw.* **12**, 154-161, doi:10.1145/6497.214326 (1986).
18 Clarkson, D. B., Fan, Y. & Joe, H. A Remark on Algorithm 643: FEXACT: An Algorithm for Performing Fisher's Exact Test in r x c Contingency Tables. *ACM Trans. Math. Softw.* **19**, 484–488, doi:10.1145/168173.168412 (1993).
19 Benjamini, Y. & Hochberg, Y. Controlling the False Discovery Rate: A Practical and Powerful Approach to Multiple Testing. *J. R. Stat. Soc. Ser. B-Stat. Methodol.* **57**, 289-300, doi:10.2307/2346101 (1995).
20 Kanehisa, M., Goto, S., Sato, Y., Furumichi, M. & Tanabe, M. KEGG for integration and interpretation of large-scale molecular data sets. *Nucleic Acids Res.* **40**, D109-114, doi:10.1093/nar/gkr988 (2012).
21 Subramanian, A. *et al.* Gene set enrichment analysis: a knowledge-based approach for interpreting genome-wide expression profiles. *Proc. Natl. Acad. Sci. U.S.A.* **102**, 15545-15550, doi:10.1073/pnas.0506580102 (2005).
22 Väremo, L., Nielsen, J. & Nookaew, I. Enriching the gene set analysis of genome-wide data by incorporating directionality of gene expression and combining statistical hypotheses and methods. *Nucleic Acids Res.* **41**, 4378-4391, doi:10.1093/nar/gkt111 (2013).
23 Best, D. J. & Roberts, D. E. Algorithm AS 89: The Upper Tail Probabilities of Spearman's rho. *Appl. Stat.* **24**, 377–379 (1975).
24 Spizzo, R., Nicoloso, M. S., Croce, C. M. & Calin, G. A. Snapshot: microRNAs in cancer. *Cell* **137**, 586-586, doi:10.1016/j.cell.2009.04.040 (2009).

25 Corcoran, C., Friel, A. M., Duffy, M. J., Crown, J. & O’Driscoll, L. Intracellular and extracellular microRNAs in breast cancer. *Clin. Chem.* **57**, 18–32 (2011).
26 O’Day, E. & Lal, A. MicroRNAs and their target gene networks in breast cancer. *Breast Cancer Res.* **12**, doi:10.1186/bcr2484 (2010).
27 Chen, P. S., Su, J. L. & Hung, M. C. Dysregulation of microRNAs in cancer. *J. Biomed. Sci.* **19**, doi:10.1186/1423-0127-19-90 (2012).
